# Carbon Nanotubes in Cement—A New Approach for Building Composites and Its Influence on Environmental Effect of Material

**DOI:** 10.3390/molecules29225379

**Published:** 2024-11-14

**Authors:** Teobald Kupka, Natalina Makieieva, Paweł Świsłowski, Małgorzata Rajfur, Artur Małolepszy, Leszek Stobiński, Stefania Grzeszczyk, Krystian Jurowski, Adam Sudoł, Roman Wrzalik, Oimahmad Rahmonov, Krzysztof Ejsmont

**Affiliations:** 1 Faculty of Chemistry and Pharmacy, Opole University, 48, Oleska Street, 45-052 Opole, Poland; makieievium@gmail.com; 2 Institute of Biology, University of Opole, 22, Oleska Street, 45-032 Opole, Poland; pawel.swislowski@uni.opole.pl (P.Ś.); rajfur@uni.opole.pl (M.R.); 3 Faculty of Chemical and Process Engineering, Warsaw University of Technology, 1, Waryńskiego Street, 00-645 Warsaw, Poland; artur.malolepszy@pw.edu.pl (A.M.); lstob50@hotmail.com (L.S.); 4 Faculty of Civil Engineering and Architecture, Opole University of Technology, 48, Katowicka Street, 45-061 Opole, Poland; s.grzeszczyk@po.edu.pl (S.G.); k.jurowski@po.edu.pl (K.J.); 5 Department of Technical Sciences, University of Applied Sciences in Nysa, 5 Obrońców Tobruku, 48-300 Nysa, Poland; adam.sudol@pans.nysa.pl; 6 A. Chełkowski Institute of Physics, University of Silesia, 75 Pułku Piechoty 1, 41-500 Chorzów, Poland; rwrzalik@gmail.com; 7 Institute of Material Science, University of Silesia, 75 Pułku Piechoty 1a, 41-500 Chorzów, Poland; 8 Institute of Earth Sciences, Faculty of Natural Sciences, University of Silesia in Katowice, 60, Będzińska, 41-200 Sosnowiec, Poland; oimahmad.rahmonov@us.edu.pl

**Keywords:** multi-walled carbon nanotubes, superplasticizer, homogenization, FT-IR, Raman, DFT, intermolecular interaction, mosses, vitality

## Abstract

An addition of carbon nanostructures to cement paste is problematic due to the difficulties in obtaining homogenous mixtures. The paper reports on a more effective way of mixing carboxylated multi-walled carbon nanotubes (MWCNT-COOH) in cement pastes. The additional biological impact of the studied nanomodified cement was analyzed in the case of two moss species’ vitality. The applied approach of obtaining a homogeneous mixture is based on intense mechanochemical mixing of MWCNT-COOH together with polycarboxylate superplasticizer (SP). As a result, a more homogenous suspension of MWCNT-COOH within a liquid superplasticizer, suitable for addition to hydrophilic cement paste, was obtained. FT-IR/Raman spectroscopy was used for materials’ characterization. To explain the mixing process at the molecular level, systematic theoretical studies using density functional theory (DFT) were performed. The structures, interaction energies and IR/Raman vibrational spectra of model carboxylic acids, mixed with functionalized SWCNTs as simplified models of real MWCNTs, were obtained. Due to the controversial opinions on the environmental hazards of carbon nanostructures, additional in vivo studies were performed. In this case, effects of cement modified by the addition of small amounts of MWCNT-COOH with SP in comparison to the composite without carbon nanostructures and control subsoil on the vitality of mosses *Polytrichum formosum* and *Pseudoscleropodium purum* were studied.

## 1. Introduction

Properties of current composites based on cement are improved by the addition of small amounts of various nanoparticles. However, the effect of these additives on the physico-chemical properties of the high-performance final product is critically dependent on the applied particles’ and nanoadmixtures’ homogeneous distribution within the mix, e.g., without their agglomeration [1].

There are several methods leading to homogeneous nanoparticle distribution without agglomeration. For example, the parallel use of efficient superplasticizers, sonification and fast mixing in cement mortar was reported [2]. However, mechanical mixing applied for degradation of the formed agglomerates of nanoparticles is not efficient [3]. The introduction of nanoparticles to concrete mix in the form of water suspension with the application of sonification is also an efficient way [4]. A detailed analysis of the effect of MWCNT nanoparticles’ addition on mechanical properties of cement matrix was reported [5]. For this reason, the design of new mixing methods is an important question in the production of material with better qualities. From the above studies, it is apparent that the addition of surfactants, which decrease surface tension and additionally show hydrophilic properties, supporting dispersion of partly hydrophobic MWCNT particles, is essential for cement paste.

It is known that the introduction of carbon nanotubes and other ordered carbon materials into the cement mix and the preparation of homogeneous mixture are difficult due to their agglomeration as well as transposition and annihilation between some clusters and/or cells, strain–stress and inner surface topography. This process is partly controlled by the formation of hydrogen bonds and, to some extent, the van der Waals interactions between the functionalized nanocarbon particles [6] and therefore an efficient dispersion of carbon nanoparticles in cement composites is necessary. This is achieved by using sonification and applying surfactants, for example, sodium dodecyl benzene sulfonate [7], sodium dodecyl sulfate [8], or an acid treatment [9], leading to functionalization of nanocarbon structures with polar groups containing oxygen (including -OH and -COOH) [10].

There have been many studies on the formation of a covalent bond between MWCNTs and various substituents, which provides changes in material hydrophilicity [9,11]. For example, groups like -OH, -COOH, -NH_2_ and grafted long acryl chains were permanently introduced to make carbon nanomaterials more hydrophilic, thus improving their mixing and dispersion in water [12]. In our earlier reports [13,14] we analyzed theoretically the structure and properties of functionalized single-walled carbon nanotubes (SWCNTs). On the other hand, experimental nanocarbon characterization using vibrational spectroscopy, including infrared (IR) and Raman techniques, is widely used [15,16]. There are several basic works on the IR technique, as well as on its application for characterization of organic systems including polymers [17,18,19,20,21,22,23]. Raman spectroscopy of single- and multi-walled carbon nanotubes, graphite and graphene, including graphene oxide (GO) and reduced graphene oxide (rGO), is also well documented [24,25,26,27,28,29,30,31,32,33,34]. Due to the polymeric nature of the used superplasticizers, their structure can be analyzed using IR and Raman techniques. Contrary to Raman studies, the presence of water nearly completely excludes IR measurements. On the other hand, a versatile nuclear magnetic resonance (NMR) technique is suitable for the study of polymers in water.

In the current study, we use a molecular modeling approach in order to obtain a deeper insight into the role of small nanocarbon additions, including functionalized MWCNTs, leading to the production of a homogenous nanocarbon suspension with superplasticizer. This could finally lead to obtaining cement materials with improved properties. The most important stage of the addition of carbon-containing (and other materials) nanofiller to cement paste is related to homogenous suspension preparation of functionalized MWCNTs or graphene in water. This is due to the fact that side walls of carboxylated nanotubes are mainly hydrophobic and their ends are functionalized with polar -COOH and -OH groups [15,16]. It is also known [15,16] that the amount of sidewall functionalization is much smaller than at their terminals. Thus, we concentrated on preparation of a nanocarbon suspension in superplasticizer for subsequent mixing with cement. In fact, the superplasticizer acts as a kind of surfactant for carbon nanotubes [35].

**Figure 1:**
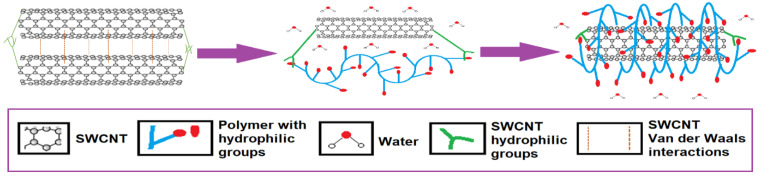
Schematic diagram explaining the interaction between polar groups of superplasticizer and carbon nanotubes, partly covered with carboxylic (and some hydroxyl) groups. Left, middle and right schemes show three stages of interaction between superplasticizer and carbon nanotube.

It is known that hydrophobic interactions promote agglomeration of several nanotubes and decrease their ability to mix with water. Additionally, as a result of strong hydrogen bonding, their ends could also stick together and hinder mixing with water [36]. However, the addition of polymeric superplasticizer with several polar groups could lead to direct H bond interactions with nanotube “hydrophilic hot spots”. Thus, the presence of superplasticizer in the liquid phase enables formation of strong interactions between MWCNT-COOH and long polymer chains, which additionally could wrap around the carbon nanotubes and thus improve their mixing with water. This process is schematically shown in Figure 1. A similar wrapping of long chains of polysaccharide (amylose) around carbon nanotubes was observed earlier [36,37]. The beneficial role of polymeric surfactants with long hydrophobic chains and polar heads in improving dispersion of carbon nanotubes was reported [35]. In a schematic cartoon, the authors [35] nicely explained the surfactant action by utilizing non-covalent dispersion interactions between non-polar surfactant chains with the hydrophobic side of carbon nanotubes. We want to admit that in the current work we do not study markedly weaker dispersion forces or interactions with -OH groups because we postulate a significantly stronger interaction between hydrophilic -COOH and COO- groups of the superplasticizer and “sensitive spots” of functionalized nanotubes. These non-covalent interactions should result in better dispersion of carbon nanomaterials in water (see Figure 1).

In the current study, commercial polycarboxylate superplasticizer and MWCNT-COOH [38,39] were characterized by Fourier transform infrared (FT-IR) and Raman spectroscopy. To explain the mixing process mentioned above at the molecular level, we decided to use theoretical modeling within a framework of density functional theory (DFT [40,41,42]). Another important aspect of the introduction of carbon nanostructures into building materials is environmental safety of the final composite. There are controversial opinions on nanomaterials’ effect on the vitality of different living organisms. Some studies demonstrate a negative influence of high concentrations of nanomaterials, but negligible changes are observed for smaller amounts of graphene-based materials [43,44]. For this reason, a biological study was carried out, analyzing the effect of pure cement paste and a paste modified by the addition of MWCNT-SP on the photosynthetic activity of living organisms, e.g., two species of mosses. These plants, as pioneer organisms, could be used to colonize anthropogenic areas and combat the so-called “concretemania”.

Mosses (Bryophyta) are small eukaryotic non-vascular plants, which usually have no internal transport system. They have a flowerless life cycle and form rhizoids as a primitive analogue of a “true” root system. As pioneer organisms, mosses occupy urbanized areas, as well as places where higher plants cannot survive due to high temperature (desert), altitude or limited light (tundra) [45]. High resistance to stressful living conditions makes mosses good candidates for biomonitoring of environmental pollution. The main advantages of using these organisms as bioindicators are a lack of crust, i.e., moss tissues are readily permeable to micro- and macronutrients, high cation exchange capacity and easy plant material availability (their colonies grow relatively fast). Other advantages of studies with moss research material are their low cost and short duration of experiments [46]. However, there are some complications with biomonitoring using mosses. Colonization of these organisms is dependent on various factors such as humidity [47]. An example is green roofs, where the depth of the substrate and the age of the roof have a dominant influence on the structure of the plant communities on it. Thin substrates and young roofs support sedum and moss species [48]. The moss layer transfer technique significantly increases the presence of moss species that were introduced during restoration. The presence of forest bryophyte species in spreading material increases their biomass in the restoration site, even on a thick residual *Sphagnum* peat layer [49]. Another use for mosses in urban areas is urban greening. They absorb and retain water, reducing surface water run-off, thus protecting the area from flooding and drying [50,51]. Due to the high availability of mosses in the urban environment they could be good model organisms for studying nanomodified materials’ harmful effects. For this reason, it seems to be useful to investigate the effects of MWCNT-modified cement paste on the viability of the mosses *Polytrichum formosum* Hedw. and *Pseudoscleropodium purum* (Limpr) M. Fleisch. ex Broth. These species are often found in European forests and could be transplanted, for example, into urban spaces.

## 2. Results and Discussion

FT-IR studies of carbon nanotubes and graphenes are extremely challenging due to the presence of tightly bound water and the obtained spectra are noisy, broad and nearly featureless. For brevity, an example of FT-IR spectra of MWCNTs is shown in Figure S1 in the Supplementary Materials.

### 2.1. Superplasticizer Characterization by FT-IR Spectroscopy

**Figure 2:**
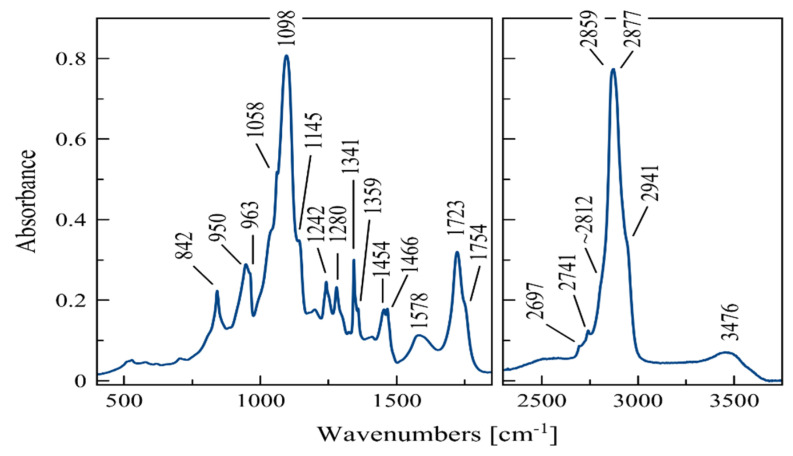
Selected fragments of the ATR spectrum of superplasticizer (SP) with peaks labeled. Partly overlapped peaks are clearly visible.

**Figure 3:**
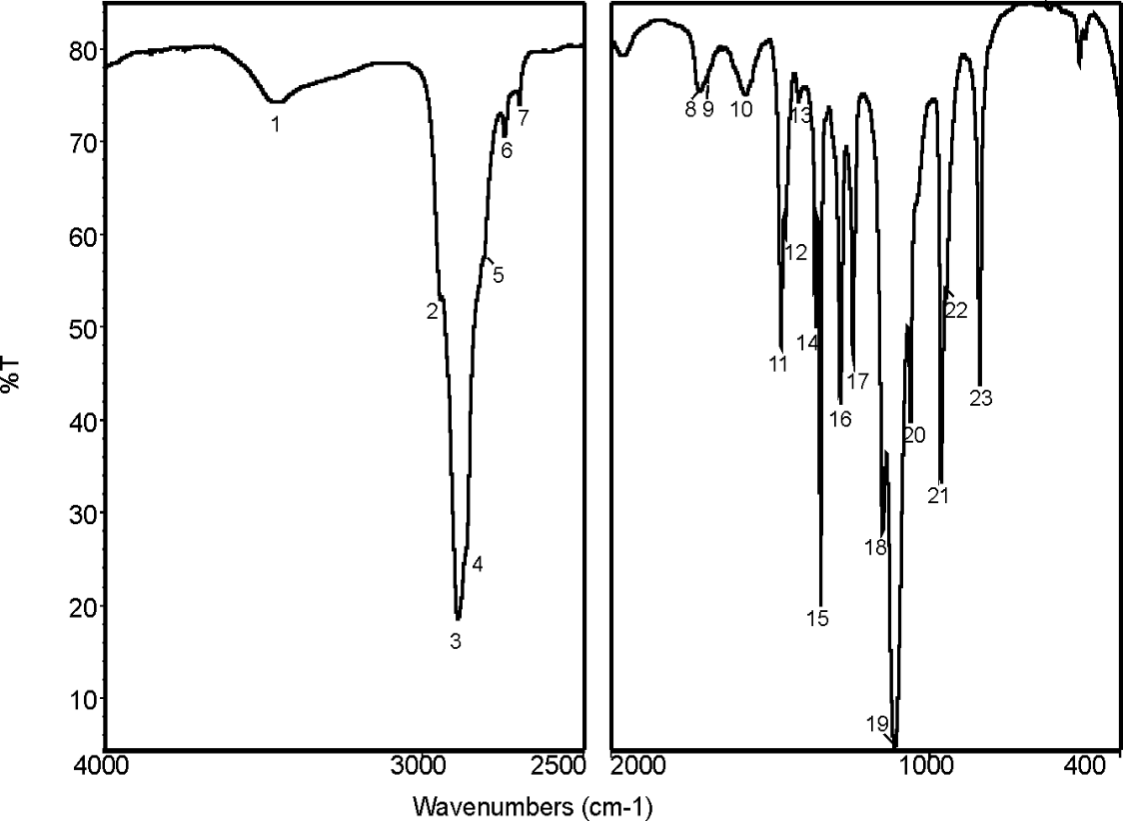
Selected fragments of the FT-IR spectrum of superplasticizer with peaks labeled.

In the first step, the applied superplasticizer was characterized using ATR and infrared spectroscopy (see Figure 2 and Figure 3). On the other hand, the ATR spectrum (see Figure 2), which does not need sample preparation before measurement, included the main spectral features preserved though some peaks were overlapped and shifted by a few wavenumbers. The observed peaks of the superplasticizer from Figure 3 were tentatively assigned and are gathered in Table 1.

**Figure 4:**
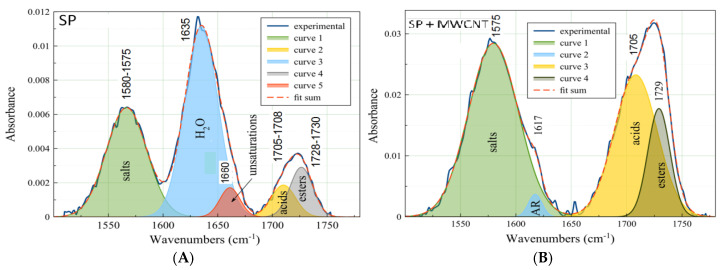
Carbonyl region fragment of FT-IR spectrum of (**A**) neat SP and (**B**) SP containing 3% MWCNT and the result of line fitting is also shown.

The carbonyl stretching fragment (1800–1500 cm^−1^) of the FT-IR spectrum with partly overlapped peaks, shown in Figure 3, was carefully analyzed and the individual peaks were labeled. In order to assign more precisely the type of functional groups containing a carbonyl fragment on the superplasticizer FT-IR spectrum, a digital line fitting procedure (deconvolution) was applied. As a result, the positions of partly overlapped signals were determined. Examples of original spectral fragments and the resolved individual bands of neat superplasticizer alone and in the presence of 3% MWCNT suspension are shown in Figure 4A (left) and Figure 4B (right), respectively. As result, it was possible to observe quantitatively the presence of carboxylic acids (-COOH at 1705 cm^−1^), esters (-COOR at 1728 cm^−1^) and ionic salts (-COO^−^ Me^+^ at 1575 cm^−1^) as fragments of SP molecules (Figure 4A (left)). In addition, it was apparent from Figure 4A that the strong band at 1635 cm^−1^ was due to the HOH bending mode of water. This band is missing in Figure 4B. In principle, it could originate from moisture present in KBr while the band at about 1660 cm^−1^ could originate from unsaturated and conjugated C=C groups, possibly from MWCNTs.

A simplified chemical structure of superplasticizer, as deduced from the analyzed IR spectra (compare Figure 2 and Figure 4 and Table 1), indicates the presence of three main molecular fragments (building blocks):

(1) Acids (R-COOH),
(2) Salts (R-COO^−^),
(3) Esters (R-COOR’).

The surfactant properties of SP are due to its specific chemical structure formed by the building blocks. Thus, the superplasticizer molecule is formed by long backbone chains containing aliphatic methylene (-CH_2_-) and methine (-CH-) groups, e.g., -CH_2_-CH-CH_2_-CH- fragments, and long side oxyethylene chains (-CH_2_-CH_2_-O-)_n_. Thus, it resembles a comb polymer structure, analyzed theoretically in solid polymer electrolytes with hopping lithium cation [52]. The main backbone chain of SP is non-polar while the side chains are highly hydrophilic, in particular due to their carboxylic terminal groups. Therefore, the applied SP shows the desired surfactant properties.

A detailed analysis of the measured infrared spectra of the applied superplasticizer (see results in Figure 3 and Figure 4 and Table 1) allows the conclusion that its molecules contain methylene groups (CH_2_) belonging to the backbone chain which are visible at about 2862 cm^−1^. These moieties are formed by saturated polyacrylic fragments (-CH_2_-CH-CH_2_-CH-)_n_ and -CH_2_- units, directly bonded to oxygen atoms (-CH_2_-O-) in side polyoxyethylene chains (see CH stretch vibrations at about 2944 and 2888 cm^−1^). In fact, the strongest band in the IR spectrum of SP at 1110 cm^−1^ belongs to polyetheric fragments -CH_2_-O-CH_2_-. Its high intensity suggests the presence of a very long polyetheric (-CH_2_-CH_2_-O-) chain, containing dozens of units. By comparing the intensity of this C-O-C stretch vibration with earlier reports [53,54], one could assume the presence of over 80 units forming the side chain. Obviously, the non-polar, hydrophobic backbones of the superplasticizer polymer will have an affinity for the non-polar, hydrophobic surfaces of carbon nanoparticles. On the other hand, polar and strongly hydrophilic carboxylate groups of the sodium acrylate ionic salt -COO^−^ Na^+^ and long hydrophilic poly(oxyethylene) chains will show affinity to the water environment of the cement mix.

The structure elements discussed above and overall properties of superplasticizer could lead to better dispersion of carbon nanoparticles in the aqueous environment of cement mix.

### 2.2. Raman Spectroscopy of Neat Superplasticizer and with Added MWCNTs

**Figure 5:**
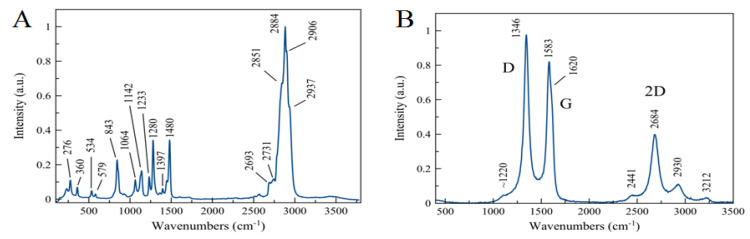
The Raman spectra of (**A**) neat superplasticizer and (**B**) SP + 3% MWCNTs.

The Raman spectra of neat superplasticizer, as well as with very small admixtures of MWCNTs, are shown in Figure 5 and the tentative signal assignment is presented in Table 1.

Raman spectra of the original superplasticizer (sample A) and with the addition of 3% MWCNTs (sample B) are shown in Figure 5. It is also worth mentioning a significant increase in the viscosity and fluorescence of superplasticizer containing very small amounts of carbon nanomaterial. Thus, it was challenging to analyze such Raman spectra and was necessary to apply baseline correction and line fitting procedures first.

The IR and Raman data shown above provide complementary information. In particular, no MWCNTs are visible from IR data in samples containing small amounts of nanostructured carbon added to superplasticizer. However, Raman spectra in Figure 5 (sample B) clearly show characteristic D and G bands of nanostructured carbon materials at about 1346 and 1580 cm^−1^. A similar ratio of D/G Raman bands was recently reported in cement paste containing a small amount of MWCNTs [55]. From the magnitude of the D band (D > G) we can conclude about the presence of significant structural disorder in the applied MWCNTs. In addition, in the range of 2440 to 3200 cm^−1^ several overtones and combination bands are observed due to the presence of carbon nanomaterial. In addition, Raman spectra of neat SP (sample A in Figure 5) reveal large signals due to CH and CH_2_ stretch modes in the range of 2850 to 2940 cm^−1^. This could be due to a repeated motif of methylene groups in oxoethyl- chains of superplasticizer, forming a kind of comb polymer.

In Table 2 the observed bands of superplasticizer (sample A) and SP in the presence of 3% MWCNTs (sample B) are gathered and the tentative peak assignments given. The apparent lack of superplasticizer, as observed from Raman spectra of sample B, seems to be confusing (see Table 2). On the other hand, very small amounts of carbon nanotubes (only 3% MWCNTs) are clearly visible (see characteristic D, G bands and their overtones and combination bands). However, very similar spectra of carbon materials in polymers were reported in the literature [56,57,58].

### 2.3. Theoretical Modeling of Superplasticizer Interaction with Carbon Nanotubes

In order to model structural fragments of superplasticizer, carbon nanotubes and their mutual interactions, we decided to select two density functionals, BLYP and B3LYP, and check their performance on several small molecules.

#### 2.3.1. Performance of BLYP, B3LYPD3BJ and BLYPD3BJ Density Functionals in Predicting Structure, Interaction Energy and Vibrational Parameters of Water Monomer and Dimer

Due to its small size, the water monomer is a convenient model for verifying the performance of lower-level theoretical methods, including DFT, with respect to benchmark CCSD(T) calculations [59]. The latter method is too expensive and will be used here only for comparison. In addition, the current theoretical results on water monomer could be directly compared with experiments in the gas phase [60], as well as with high-level theoretical calculations at the coupled cluster level of theory [61]. It is known that BLYP and B3LYP density functionals are able to model geometry and energy of hydrogen bonds, which are dominated by electrostatic interactions. However, to account for long-range dispersion forces, also present as small components of H bonds, the use of empirical terms modeling these interactions, for example, the GD3BJ term, introduced by Grimme, is recommended. In our studies, we were interested in recovering the total electrostatic and dispersion effects and we applied the B3LYPD3BJ and BLYPD3BJ functionals. In addition, small carboxylic acids were selected as models of superplasticizer structure fragments. The possibility of carboxylic groups interacting with sodium and calcium cations and with functionalized carbon nanotubes was also taken into account.

For brevity, in Table S1 in the Supplementary Materials, geometrical and vibrational parameters of water monomer are compared, predicted by BLYP, B3LYPD3BJ and BLYPD3BJ density functionals, combined with a large and flexible aug-cc-pVTZ basis set against experiment and benchmark CCSD(T) results [60,61]. It is apparent that the selected density functionals predict very accurate OH bond length and HOH angle of the water monomer which deviate from experiments by 0.050–0.015 Å and 0.04–0.6°, respectively. Additionally, the structure of water, predicted without and with the D3BJ dispersion term, is the same. As expected, the benchmark coupled cluster method, combined with a large basis set [61], reproduces the experiment very well.

It is known [62,63,64,65] that in routine molecular modeling calculations, the predicted harmonic frequencies are not computationally expensive but, due to omission of anharmonicity, they significantly overestimate experiment. However, it is apparent from Table S1 that the water monomer wavenumbers of symmetric and asymmetric OH stretch and HOH deformation modes are very accurately predicted in both high-level harmonic and anharmonic CCSD(T) calculations (RMS < 3 cm^−1^). On the other hand, B3LYPD3BJ/aVTZ calculations also produce acceptable and fairly accurate wavenumbers (RMS < 35 cm^−1^). The corresponding BLYPD3BJ/aVTZ results are about five times worse. However, when the latter results are “treated” as fundamental ones, they reproduce experimental results extremely well (RMS ~1 cm^−1^).

Prediction of water dimer geometry and interaction energy allows both the determination of forces, which keep two H bonded monomers together, and its molecular structure. However, with limited size basis sets, one has to take care about the basis set superposition error (BSSE [66]), for example, by applying the counterpoise method (CP [67]).

The results of unconstrained optimization of linear water dimer, which is the most stable one [68,69,70,71,72,73,74], using BLYPD3BJ and B3LYPD3BJ density functionals, combined with the aug-cc-pVTZ basis set, are included in Figure S2. It is apparent that the geometrical parameters calculated with both density functionals are very similar but the counterpoise-corrected interaction energies, calculated with the former method, are significantly closer to the benchmark CCSD(T) result (−4.94 vs. −5.02 kcal/mol [68], see Table S2). Very accurate BLYPD3BJ interaction energies of H bonded dimers (close to CCSD(T) results) in comparison to B3LYPD3BJ were also reported by Řezáč [75].

Apart from interaction energy, in Table S2 are also gathered structural and vibrational parameters of water dimer, calculated with BLYPD3BJ and B3LYPD3BJ density functionals and using the aug-cc-pVTZ basis set and compared with the available experimental and benchmark coupled cluster results [65,68,69,76,77,78,79,80]. Obviously, the distances calculated with BLYPD3BJ are somewhat longer than the corresponding B3LYPD3BJ ones but still very accurate. It is also evident from Table S2 that both CCSD(T) and DFT methods predict vibrational frequencies with lower accuracy than structural parameters. However, the B3LYPD3BJ predicts water dimer wavenumbers about three times better than BLYPD3BJ. Additionally, one has to remember that to obtain better agreement with the observed IR/Raman data, the theoretical vibrations are often scaled [81].

#### 2.3.2. Monomer and Dimer Properties of Formic and Acetic Acids

The next two briefly tested model molecules are formic and acetic acids. These compounds have been studied using both experimental and theoretical methods [82,83,84,85,86,87,88,89,90,91,92,93,94,95,96,97,98,99,100,101,102,103,104,105,106,107,108]. The BLYPD3BJ and BLYP optimized structures of *trans* conformers of carboxylic acids, which are the most stable ones, are shown in Figure S3. It is apparent from Table S3 that both BLYPD3BJ- and BLYP-calculated formic and acetic acid bond lengths very accurately reproduce experiment and benchmark theoretical results (RMS deviations of 0.008–0.022 Å, see [83,85,86,89,90,91,105]). In particular, the B3LYP results closely reproduce CCSD(T) data. However, as expected, these results are somewhat worse than those obtained from very expensive computations at both CCSD and CCSD(T) levels of theory [86,109].

The selected level of DFT theory (BLYP/aVTZ and BLYPD3BJ/aVTZ) is also able to predict fairly accurate harmonic vibrations of formic acid monomer [86,89,91,105,110] and the RMS deviations of about 31 cm^−1^ with respect to experimental IR and Raman data, as well as benchmark coupled cluster results, are relatively small (see Table S4). However, the corresponding VPT2-calculated RMS of about 96 cm^−1^ is very bad. Interestingly, the harmonic vs. anharmonic values for B3LYP (and B3LYPD3BJ) show the opposite trend (Table S4C, RMS of 68 and 22 cm^−1^). It is apparent from Table S4 that the harmonic BLYP frequencies are over two times better than the B3LYP ones.

However, the accuracy of theoretical interatomic distances and vibrational frequencies of formic acid monomer is lower than in the case of water monomer (see Tables S3 and S4). On the other hand, the anharmonic (VPT2) frequencies calculated by BLYP and BLYPD3BJ density functionals are unacceptable (RMS of ~100 cm^−1^, see Table S4B).

**Figure 6:**
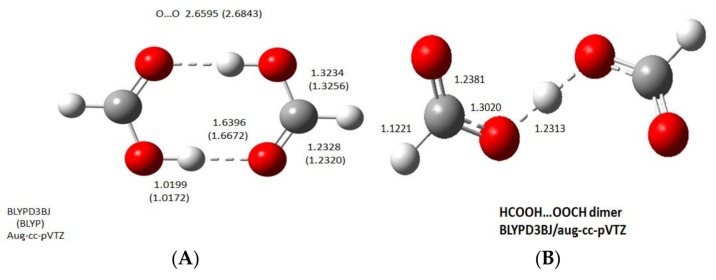
BLYPD3BJ- and BLYP-optimized structures of (**A**) *trans*-formic acid dimers and “mixed dimer HCOOH⋯OOCH” (**B**) (interatomic distances are in Å).

The BLYP-optimized structure of a typical formic acid dimer, formed by two neutral formic acid molecules, together with indicated bond lengths without and with inclusion of dispersion, is shown in Figure 6A. The cyclic dimer is held by two BLYP- and BLYPD3BJ-estimated H bonds differing by only ~0.03 Å.

The other “mixed” formic acid dimer is formed by its anion and a neutral molecule (Figure 6B). The BLYPD3BJ- and B3LYPD3BJ-calculated interatomic distances are also shown in the scheme. Very short H bonds indicate strong interactions.

As before, both BLYPD3BJ and BLYP density functionals predict similar (but not identical) formic acid dimer interatomic distances (see Figure 6A and Table S6) and very accurately reproduce coupled cluster [86] and experimental results [90,105] (deviation of 0.01–0.03 Å). However, there is a significant difference in the optimized structure shown in Figure S2B, predicted by BLYP and BLYPD3BJ density functionals. In other words, upon inclusion of dispersion effects, the H atom is more localized at one formate molecule and the other shows a more pronounced carboxylic anion structure. Thus, the former functional predicts the carboxylic H atom exactly in the middle between two oxygen atoms (H⋯O of 1.2313 Å) and the O⋯O separation is 2.4574 Å. The latter density functional produces an asymmetric H bond with the H atom localized closer to one oxygen atom (O⋯H distances are 1.1734 and 1.2974 Å). Additionally, the O⋯O distance is slightly shorter (2.4654 Å).

There are 24 normal modes of formic acid dimer [86,94,95,96,110]. Both density functionals predict its vibrational frequencies reasonably well with RMS deviations from 20 to 40 cm^−1^ (Tables S7 and S8). However, the inclusion of an empirical dispersion term slightly deteriorates the accuracy of the predicted vibrations.

A very similar structure to formic acid dimer was obtained for acetic acid dimer (Figure S4), too. The corresponding harmonic and anharmonic frequencies for acetic acid dimer, calculated with BLYP and B3LYP density functionals (with and without the GD3BJ term), are gathered in Table S9. In this case, B3LYP predicts some anharmonic vibrations higher than the corresponding harmonic ones.

It is known that both formic and acetic acids form very strong dimers which are also present in the gas phase [93]. Thus, it was interesting to calculate the corresponding interaction energies holding two monomers in a dimer (see Table 3).

The benchmark interaction energy for *trans*-formic acid dimer calculated at the CCSD(T)/CBS level of theory is −18.753 kcal/mol [94,112,113]. From the energy difference between BLYP and BLYPD3BJ results, it was possible to see a significant contribution of dispersion in the total interaction energy (14 and 20% for formic and acetic acid dimers). It was observed that the accuracy of structure optimization has a small effect on the value of the interaction energies. Higher-level theory interaction energies, such as aug-cc-pVTZ and aug-cc-pV5Z, differ only a little. It is worth noticing that our BLYP result for formic acid dimer is close to benchmark coupled cluster results. Interestingly, for the applied aug-cc-pVTZ basis set, a fairly small BSSE was calculated (about 0.25–0.30 kcal/mol). Single-point CCSD(T) calculation with a very large basis set (aug-cc-pV5Z) indicated a very small basis set superposition error (0.02 kcal/mol).

#### 2.3.3. Performance of BLYP, B3LYPD3BJ and BLYPD3BJ Density Functionals in Predicting Structure, Interaction Energies and Vibrational Frequencies of Formic and Acetic Acid Salts with Na^+^ and Ca^2+^

**Scheme 1:**
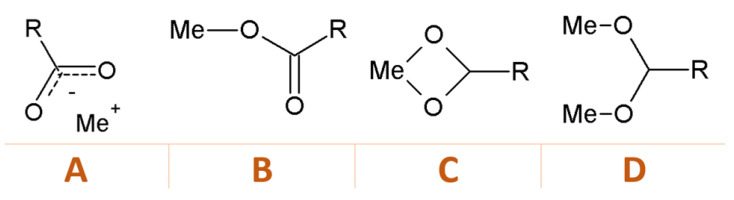
Observed patterns for metal carboxylates: (**A**) ionic systems and (**B**–**D**) with more covalent and coordinated character.

Molecules of superplasticizer could strongly interact with polar groups of carbon nanotubes forming typical carboxylic dimers R-COOH …. HOOC-R’ or partly ionized R-COOH⋯(OOC-R)^−^ ones. Obviously, the presence of metal cation Me^+^ will lead to differently charged structures like R-COO-Me^+^-OOC-R. The structure patterns of metal carboxylates depend on the type of cation [114] and the dominating structures for sodium and calcium are structures A and C, respectively (see Scheme 1).

In our systems, both Na^+^ and Ca^2+^ cations are present [114]. First, the compounds containing formic and acetic acid dimers without coordinated water or hydrated dimers are considered.

**Figure 7:**
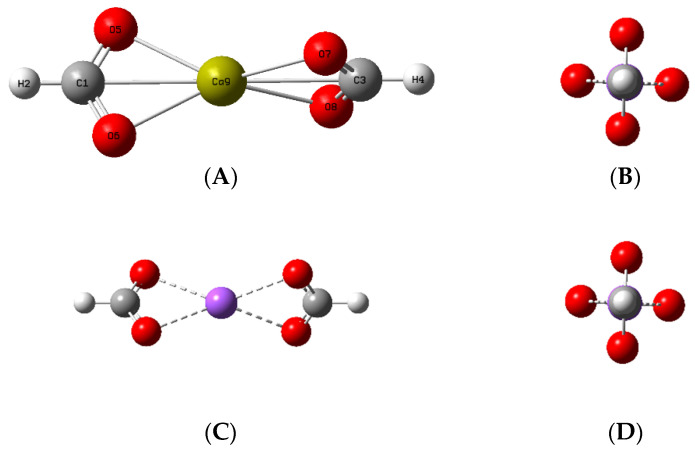
Side (**A**) and top (**B**) view of BLYPD3BJ/aug-cc-pVTZ structure of calcium (neutral complex) and sodium formate ((**C**,**D**), total charge = −1).

Anhydrous calcium and sodium formates (Figure 7) contain two perpendicular COO^−^ groups. This arrangement of atoms allows minimization of repulsion between negative oxygen atoms.

In our systems containing superplasticizer, carbon nanotubes and cement components, both Na^+^ and Ca^2+^ cations are present. In the subsequent stage of modeling, first their anhydrous salts of formic and acetic acids and then the hydrates are considered.

Anhydrous calcium and sodium formate dimers (Figure 7) show two COO^−^ groups which are oriented perpendicularly to each other to minimize repulsion of negatively charged oxygen atoms.

**Figure 8:**
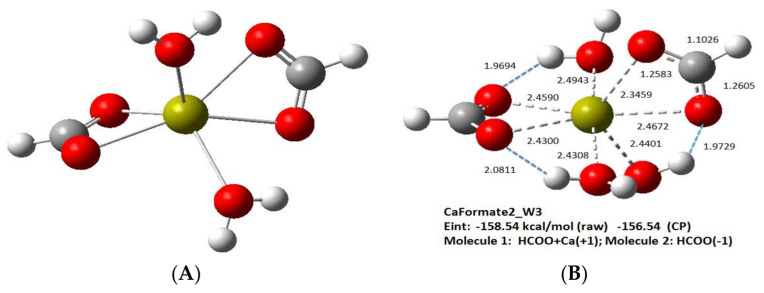
B3LYPD3BJ/aug-cc-pVTZ structure of calcium formate with (**A**) two and (**B**) three water molecules (aug-cc-pVTZ-X2C basis set for calcium).

A more realistic model of calcium and sodium formate should contain solvent in their first hydration sphere. Taking into account available coordination numbers (CNs) of calcium (from 4 to 9 [114]), we arbitrarily chose the structure with CN = 6 and 7. Therefore, three water molecules could be located in the first hydration shell. The B3LYPD3BJ/aug-cc-pVTZ-optimized structure of Ca(OOCH)_2_(H_2_O)_3_ in the gas phase is shown in Figure 8. Water molecules are additionally stabilized by a network of H bonds which also include a water donor hydrogen atom and acid oxygen as a proton acceptor (H_d_⋯O_acid_). The calculated H bond lengths are short (1.97–2.08 Å) and indicate fairly strong interactions. The separation between calcium cation and water oxygen atoms is within a range from 2.43 to 2.49 Å. A typical ionic structure with a calcium atom located between two carboxylic oxygen atoms of both formate anions is predicted with Ca⋯O distances from 2.35 to 2.47 Å.

**Figure 9:**
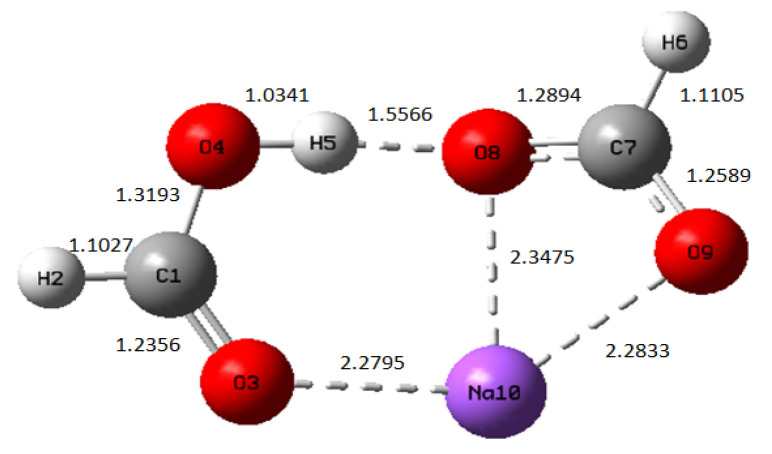
BLYPD3BJ/aug-cc-pVTZ structure of mixed sodium formate salt.

In the case of superplasticizer, which contains both protonated and deprotonated carboxylic groups and sodium cations, we could expect formation of mixed dimers and salts. Using formic acid as a simple model, structures of mixed formic acid–formate anion (Figure 8B) and mixed sodium formate salt were optimized (Figure 9). The first structure indicates a typical symmetric dimer with a hydrogen atom shared by both formate groups. The O⋯H⋯O bridges are very short and strong (1.2313 Å). Thus, the raw and CP-corrected interaction energies are −49.84 and −49.60 kcal/mol.

In the case of mixed salt, a strong H bond between the H5 atom of formic acid and the O8 atom of the formate anion is also formed (1.56 Å). The sodium cation is closely connected to three oxygen atoms (O9, O8 and O3 with separations of 2.28, 2.28 and 2.35 Å).

Calcium hydroxide is a type of small molecule, which are very important for cement chemistry. It is a linear molecule but some earlier DFT calculations reported an angle of O-Ca-O smaller than 180 degrees [115]. The B3LYP/6-311++G(3df,3pd) bonds and O-Ca-O angle are 2.038, 0.953 Å and 170^o^, respectively. However, the MP2 results indicated a linear structure with bonds of 2.046 and 0.953 Å. The mentioned discrepancies are due to the fact that Ca(OH)_2_ is a very computationally demanding chemical compound [116].

**Figure 10:**
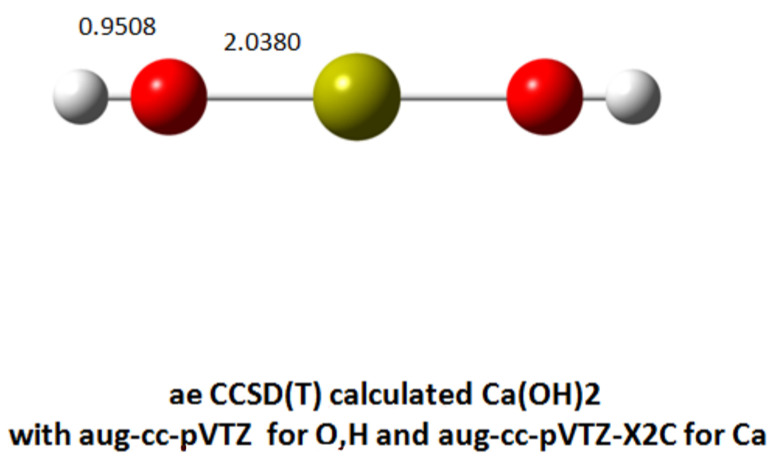
Fully optimized linear ae CCSD(T)/aug-cc-pVTZ structure of Ca(OH)_2_.

To clarify the above results, we optimized the calcium hydroxide structure using density functional B3LYP and compared it with our high-level coupled cluster results. The optimized linear structure of Ca(OH)_2_, calculated with the all-electron (*ae*) CCSD(T) method, combined with the aug-cc-pVTZ basis set for O and H atoms and aug-cc-pVTZ-X2C for Ca, is shown in Figure 10. Interestingly, in our study, the B3LYP with the same basis sets predicted very similar bond lengths (2.0298 and 0.9533 Å) of the linear molecule with all positive harmonic frequencies. Ca(OH)_2_ was additionally optimized with larger and more flexible basis sets (aug-cc-pVQZ, aug-cc-pwCVQZ and aug-cc-pwCVQZ-X2C for H, O and Ca atoms) using SCF-HF, B3LYP and MP2 methods. These calculations also resulted in linear structures with all positive wavenumbers and CaO and OH bond lengths of 2.0461 and 0.9322, 2.0298 and 0.9533, 2.0209 and 0.9526 Å, respectively.

However, the B3LYP/aug-cc-pVTZ calculations resulted in a slightly non-linear molecule with Ca-O-H and O-Ca-O angles of 179.29 and 176.88º and Ca-O and O-H bond lengths were 2.0352 and 0.9547 Å, respectively. In addition, one negative frequency was observed (−53.03 cm^−1^), indicating the presence of transition (TS) instead of a ground state. In conclusion, our DFT, MP2 and CCSD(T) values are close to benchmark CCSD(T)/aug-cc-wCVQZ results reported by Radom and coworkers (2.036 and 0.952 Å) [116].

#### 2.3.4. BLYPD3BJ Modeling of Superplasticizer Fragment Structures

A superplasticizer, in particular the polycarboxylate type, acts as the surfactant in cement paste, but it could also significantly improve mixing of nanocarbon material with water. Its action is directly related to its molecular structure, partly reflected in the corresponding IR and Raman spectra (see Figure 2 and Figure 4A).

However, due to the large size of polymeric superplasticizer molecules, we arbitrarily selected (and named) three distinct structural fragments for subsequent theoretical analysis:

(A) Acid (14 atoms),
(B) Salt (a carboxylate anion, 13 atoms),
(C) Ester (24 atoms).

**Figure 11:**
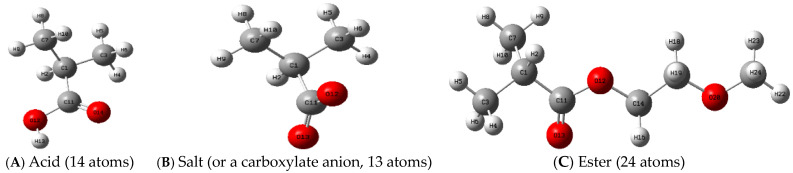
Optimized models of superplasticizer building blocks: (**A**) Acid, (**B**) Salt (or a carboxylate anion) and (**C**) Ester.

These structures contain two methyl groups representing the main polymeric chain. The molecules of acid, salt and ester shown below were fully optimized at the B3LYP/aug-cc-pVTZ level of theory (see Figure 11).

It is worth mentioning that, in recent studies, direct interactions between formic acid dimer and (6,6) and (8,8) SWCNTs using DFT and MP2 calculations were reported [108]. However, the authors modeled a small molecule (formic acid dimer) confined inside the carbon nanotube. It was observed that, in the case of intermolecular interactions with graphene-based materials, the model size had no significant effect on energy value [117]. For this reason, it is likely that the interactions of the SWCTs with small SP models will not significantly affect the quality of the obtained results and their interpretation.

#### 2.3.5. Structure of Model Zigzag (5,0) SWCNT-COOH

**Figure 12:**
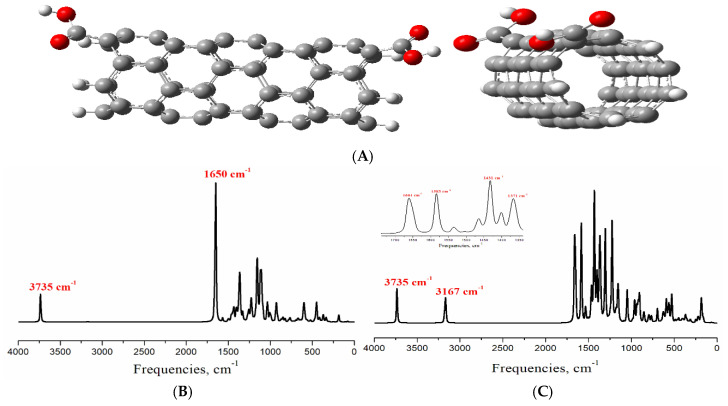
(**A**) Optimized B3LYPD3BJ/6-311++G** structure of model fragment of zigzag (5,0) SWCNT-COOH composed of five “belts” and the predicted (**B**) IR and (**C**) Raman spectra.

Due to the complexity of modeling multi-walled carbon nanotubes, a single-walled nanotube model was used in this work. It is worth noting that in the context of the interaction of the mentioned nanomaterial with a superplasticizer, hydrophilic substituents play a key role, so replacing the MWCNT by an SWCNT does not significantly affect the quality of the obtained results. In the case of fairly large carbon nanotubes, containing 76 atoms, to decrease the computational demands, we reduced the size of the basis set from aug-cc-pVTZ to 6-311++G**. Side and top views of a fully optimized B3LYPD3BJ/6-311++G** zigzag (5,0) SWCNT structure, substituted with single carboxyl groups at both ends, are shown in Figure 12. Additionally, all free valences at both ends of the SWCNT are capped with hydrogen atoms.

**Figure 13:**
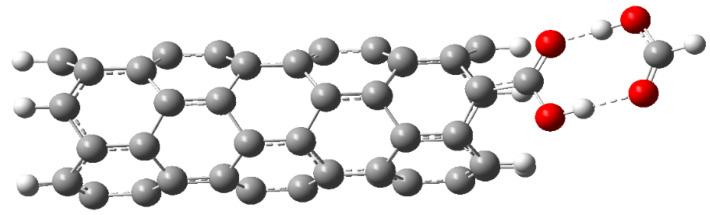
Optimized B3LYP/6-31+G* structure of model fragment of zigzag (5,0) SWCNT-COOH interacting with HCOOH.

Additionally, the corresponding theoretical IR and Raman spectra, predicted for the model SWCNT-COOH molecule, are also shown in Figure 12B and Figure 12C, respectively. Unfortunately, the quality of experimental IR spectra of SWCNT-COOH is low and not suitable for a meaningful comparison (Figure S1). For easier comparison with the experiment (see Figure 5B), the Raman spectrum in Figure S5 is plotted using 20 cm^−1^ line broadening. For brevity, the theoretical Raman spectrum of this model is also shown in Figure 12C with default line broadening of 4 cm^−1^ preserved. The theoretical Raman spectrum clearly indicates bands due to -COOH vibration at 3735 and asymmetric stretch CH at about 3267 cm^−1^. In addition, the predicted C=C vibrations at 1583 and 1371 cm^−1^ of the SWCNT model are present in the range of G and D bands observed in our recorded Raman spectra (compare Figure 4B and Figure 13). However, the contribution ratio of –COO^−^ groups in real samples is significantly smaller than that from the aromatic rings. Thus, in experimental spectra aromatic carbon fragments vibrations dominate.

#### 2.3.6. Model Zigzag (5,0) SWCNT-COOH Interaction with a Carboxylic Acid

As shown in the previous sections, the -COOH functional groups could strongly bind with polar fragments of superplasticizer and subsequently wrap it around the SWCNT (see Figure 1). Thus, in the theoretical stage of our study we modeled H bond type interaction between our model of a functionalized carbon nanotube and formic acid. The latter compound is selected as the smallest but feasible for calculation model of superplasticizer (Figure 13). Nevertheless, such calculations for a molecular system containing 76 atoms were fairly demanding. Thus, we significantly limited the basis set size and used the Pople type 6-31+G* one. As a result of the calculations, a stable structure was obtained (Figure 13).

In order to estimate the interaction energy of this model, we performed additional single-point counterpoise calculations. As expected, the raw and CP-corrected energies were about −21.98 and −20.63 kcal/mol, respectively. This result supports a strong interaction between the functionalized single-walled carbon nanotube and superplasticizer.

In real samples of superplasticizer and MWCNTs, the H bond interaction takes place for a large number of -COOH groups and the total interaction energy is significantly higher. These interactions result in the formation of stable SP-MWCNT complexes with a specific spatial arrangement. In this case, lone SP hydrophilic groups do not contribute to intramolecular bonds and become more accessible for interactions with the environment. Thus, our theoretical model supports strong interactions between SP and MWCNT-COOH that result in improved mixing with water.

### 2.4. Biological Tests

Descriptive statistics for the PSII photosynthetic activity of the two moss species are summarized in Table 4.

**Figure 14:**
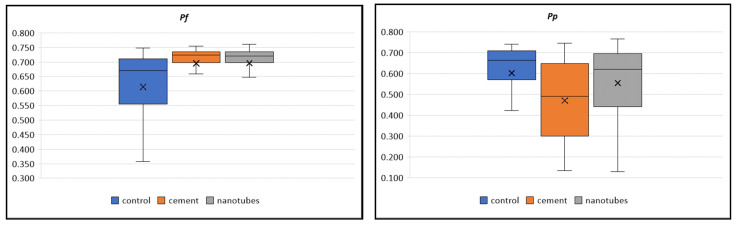
Comparison of photosynthetic activity between individual samples in the species *Polytrichum formosum* (Pf) and in the species *Pseudoscleropodium purum* (Pp).

Data presented in Table 4 show the variation in values between samples and species. Figure 14 shows substrate influence on both species’ photosynthetic activity.

The statistical significance of these values is presented in Table 5 and it shows differences between samples within and between species.

As can be seen from Table 5, data for both species within different substrates and between species show different statistical significances based on the kind of substrate. For the moss “Pf”, data from control conditions differ from the other substrate types. However, there are no statistically significant differences between cement vs. MWCNT-cement composites. In the case of Pp moss, the relationship is slightly different. In particular, the type of substrate, e.g., cement vs. MWCNT-cement, influences statistically significant differences in the PSII photosynthetic activity values of the species. The difference is also apparent from the comparison of these values between the species, where we can also see differences at the *p* < 0.001 level between cement and Pf vs. cement and Pp and carbon nanotubes and Pf vs. carbon nanotubes and Pp.

Environmental factors have an important influence on the plant (including moss) life cycle [118]. For example, the impact of temperature and light intensity can directly influence the chlorophyll content, the photosynthetic rate in mosses and thus photosynthetic activity, e.g., in peat mosses [119,120]. The type of substrate used also plays a key role in the growth and development of mosses [121]. As shown in Table 4 and Figure 14, both species, depending on the substrate, showed variable photosynthetic activity during the two-month-long experiment. The fact that the mosses maintain their vitality may be related to their entry into cryptobiosis. Throughout their life cycle they are able to vegetate in this way for a very long time [122]. The established cut-off value is 0.1 [123]. Below this value, mosses should only be considered as a natural pollutant sorbent and not as a living bioindicator and organism. As indicated by the results of our analyses, no values < 0.1 were recorded during the study period. However, there is a noticeable difference between species (see Figure 14), as confirmed by a statistically significant difference (Table 5). This indicates a variable adaptation mechanism of each species to a change in living conditions (transfer from natural ecosystem to artificial conditions). Despite the provision of relatively constant conditions for functioning (humidity, light intensity, photoperiod), the mosses were characterized by variable photosynthetic activity, which may reflect the individual response of each moss during the measurements. Differences are seen relative to the used substrates on which mosses live.

## 3. Experimental

### 3.1. Materials

**Figure 15:**
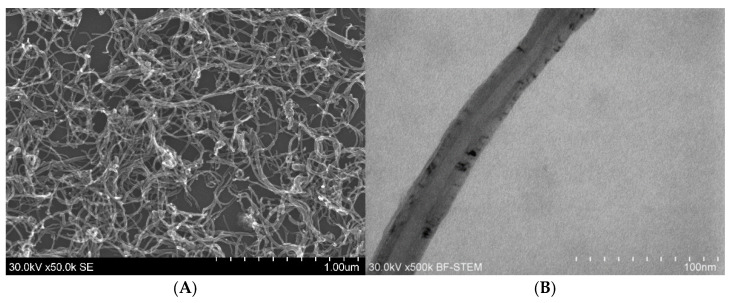
TEM image of (**A**) bundled MWCNTs and (**B**) enlarged fragment of MWCNT.

In this study, we used a commercial polycarboxylate superplasticizer (BASF, see Figure S6) (“Master Glenium ACE 420” as 30% solution in distilled water) as a surfactant to form a homogeneous mixture of multi-walled carbon nanotubes (MWCNTs). Raw multi-walled carbon nanotubes (MWCNTs) with the trade name CTUBE 100 from CNT Co., Ltd. (Suwon-si, Republic of Korea, provided in 2015) were used (see Figure 15).

The nanotubes were synthesized by thermal vapor deposition (thermal CVD) and characterized by several techniques, including TEM, SEM, elemental analysis, EDX, FT-IR, Raman spectroscopy and electron spectroscopy methods [39]. The diameter of the used MWCNTs was from 20 to 40 ± 10 nm and their length from 1 to 10 μm [39] (see Figure 15A showing TEM image of bundled MWCNTs and an enlarged fragment of a single tube in Figure 15B). Thus, MWCNTs were characterized by a large length-to-diameter ratio and their ends were carboxylated which facilitated their binding to cement grains via formation of MWCNT-COO- - - Ca^2+^- (cement grain) ionic bonding [55] and non-covalent hydrogen bonds of -COOH - - - HO- (cement grain). This leads to long, bundled “anchor” nanotubes and bridging small cracks. The used multi-walled carbon nanotubes were carboxylated (MWCNT-COOH) mainly at their ends. However, for convenience, in the following parts of the study we refer to them as MWCNTs.

Cement composites for biological tests were prepared using CEM I 42.5 R (CEM I) cement from Odra Cement Plant in Opole (Poland).

### 3.2. Biological Materials

Two moss species were used in the biological study: *Polytrichum formosum* Hedw. (Pf) and *Pseudoscleropodium purum* (Limpr) M. Fleisch. ex Broth. (Pp). Plant material was collected at the beginning of July 2024 from forests in Opole Voivodeship in the Bory Niemodlińskie region of the Prószków Forest District near the cities of Prószków and Jaśkowice (N50.581981, E17.825761 and N50.582360, E17.825151). Mosses were sampled in open areas, at least three meters from the nearest tree crown, from the ground, according to the ICP Vegetation protocol guidelines [124]. The collected samples were transported to the laboratory using the transplant method [125].

### 3.3. Methods

#### 3.3.1. Preparation of Suspensions of MWCNTs in the Superplasticizer

In general, it is not easy to prepare a uniform mixture of carbon nanomaterial and cement. As result of several tests, we decided to first make a homogeneous mixture of MWCNTs in liquid superplasticizer (see Figure S7). In other words, a kind of mechano-chemical method was used to make a homogenous mixture of partly hydrophobic carbon nanotubes in a water environment. A three-roll mill (EXAKT 80S) was used to prepare suspensions of carbon nanomaterials in the superplasticizer. The grinding process was carried out with a minimum gap of 5 μm between the rolls. This process was carried out until a homogeneous suspension was obtained (after three rolling cycles).

A suspension of MWCNTs in superplasticizer was prepared with the above method. FT-IR and Raman characterization was performed for the superplasticizer (SP) and SP + 3% wt. MWCNTs.

#### 3.3.2. FT-IR Spectroscopy

The applied superplasticizer was studied using FT-IR spectroscopy in the form of a thin liquid film between two KBr pellets. It was possible to measure samples of MWCNTs suspended in SP in the form of KBr pellets (5 mg/500 mg KBr, like for typical IR measurements of solid state samples). All FT-IR spectra were obtained in the range of 4000 to 400 cm^−1^ using a Thermo Nicolet Nexus spectrometer and with a resolution of 2 cm^−1^.

In addition, for fast characterization of the studied samples, the attenuated transmission reflectance (ATR) spectra were also recorded. The ATR spectra show the main features of FT-IR spectra though their resolution is significantly lower (close signals are more overlapped with slightly shifted peak positions).

#### 3.3.3. Raman Spectroscopy

A Raman confocal Alpha 300R microscope with an Olympus 50x/0.50 long working distance objective was used to collect spectra in the spectral window of 150–4000 cm^−1^. The laser beam with a wavelength of 532 nm and a power of 1.5 mW was focused on a small area of the sample and the Raman signal, recorded with a 1s acquisition time, was acquired 200 times.

Raman spectra were measured from small amounts of oily and very thick liquid superplasticizer and its suspension containing 3% MWCNTs.

A flat baseline and good-quality spectrum of superplasticizer (with high signal-to-noise ratio) was observed. However, the addition of MWCNTs resulted in strong fluorescence of the sample and the spectra were recorded with a highly raised baseline. Thus, to obtain meaningful spectra it was necessary to perform digital baseline correction prior to analysis.

### 3.4. Computational Details

All calculations were performed using the Gaussian 16 C.01 program [126]. Obviously, the size of a real system is too big for molecular modeling and, therefore, the selection of smaller but reasonable models is essential [62]. It was also important to choose proper theoretical tools [62]. Due to its efficiency in predicting accurate structures, energies and spectroscopic parameters, we selected DFT and applied the B3LYP hybrid density functional [42,127,128,129]. In addition, in some cases we also used an older, “pure” density functional—BLYP [127,130]. In our earlier works we noticed that this functional works somewhat faster and provides improved vibrational frequencies but predicts slightly less accurate geometry [131,132]. Models of superplasticizer and carbon nanotubes, selected for calculations, are fairly large. Thus, we decided to start an unconstrained optimization of geometry with a very small basis set, 3–21G, first and subsequently used larger ones (6-31G*, 6-311++G** and in some cases also aug-cc-pVTZ, subsequently abbreviated as aVTZ) [62,133,134,135,136]. It should be noticed that due to the presence of multiple bonds and lone electron pairs in structures of our models, the use of basis sets containing both polarization and diffuse functions is essential [62,133,134,135,136]. Additionally, to account for weak long-range dispersion interactions we also implemented the recently introduced Grimme’s empirical term GD3 [137] with Becke–Johnson dumping correction BJ [138].

In order to verify the selection of two density functionals and empirical correction of dispersion, we initially checked their performance on two small molecules and their homodimers—water and formic acid. In the next step, we modeled the structure and H bonding interactions between the two simplest protonated and deprotonated carboxylic acids and their interactions with sodium and calcium cations. Finally, we analyzed the interactions between fragments of carboxylated single-walled carbon nanotubes and fragments of superplasticizer molecule. Obviously, the use of the MWCNT model is significantly more computationally expensive and therefore it was not modeled in the current study.

### 3.5. Composite Preparation for Biological Studies

Cement composites for biological tests were prepared and their composition is shown in Table 6.

### 3.6. Biological Studies

In the laboratory, mosses were re-identified to ensure species homogeneity, using a SZ61 microscope (Olympus, Tokyo, Japan) and an IPOS-810 (Delta Optical, Gdańsk, Poland). The process was carried out in the research section of the International Research and Development Center of the University of Opole (MCBR UO). The experiment was conducted in the FITO-R phytotron rack (Biogenet Ltd., Gdańsk, Poland) in the MCBR UO building (see Figure S8). Mosses were cultured on three types of substrate: control (3 cm layer of sterile sand), hardened cement paste (3 cm layer), hardened MWCNT-modified cement paste (3 cm layer). Culture conditions, including maintaining the appropriate temperature of 21 °C and humidity of 50%, were determined according to the literature [139].

In order to assess the effect of substrate type on the viability of mosses—photosystem II chlorophyll fluorescence (PSII)—the actual photochemical productivity (yield) [140] was measured using a modulated portable fluorometer (Opti-Sciences, Hudson, NH, USA) under ambient light conditions [141]. PSII was measured three times a week for a period of two months from 12 July to 14 September 2024. For each species and each substrate variant, three measurements were taken (81 measurements). A total of 486 measurements were taken.

Statistical analysis of the results obtained was performed using Microsoft Excel 2021 and STATISTICA (version 13.3) software for data processing and visualization. For descriptive analysis, basic descriptive statistics values (min, max, median, mean with standard deviation, variance, skewness, kurtosis and sum) were calculated for photosynthetic activity data across variants (Table 4). The significance of differences between species and substrates was ensured by independent pooled sampling [142]. The normality of the data was tested using the Shapiro–Wilk test. To avoid significant differences between photosynthetic activity values leading to non-normal distribution of the data, a Box–Cox transformation was applied to improve and increase the normality of the data [143]. Lacking such improvement in terms of obtaining a normal distribution, differences between photosynthetic activity values were assessed using the non-parametric Mann–Whitney U statistical test. A difference was considered statistically significant when *p* < 0.05.

## 4. Conclusions

Mixing nanocarbon structures with cement paste to obtain homogenous mixtures is a very difficult process. It was demonstrated in the current study that a simultaneous blending of MWCNTs with polycarboxylated superplasticizer using an efficient mixing mill could produce a well-dispersed suspension, which is suitable for easier addition to cement. FT-IR and Raman spectroscopy were used to characterize a superplastisizer and suspension of MWCNTs with SP in a water environment. The experimental findings were supported by theoretical modeling of interactions between fragments of polymeric superplasticizer (acid, salt and esters) and functionalized carbon nanotubes. The density functional theory was applied to obtain structural and energetic parameters of the fully optimized models. The BLYP and B3LYP density functionals combined with reasonable size basis sets predicted reliable structures and IR/Raman vibrational spectra of interacting molecules (water, formic and acetic acids and their dimers).

Finally, the finite models of SWCNT-COOH carbon nanotubes were also built and their interaction with formic acid representing the superplasticizer was assessed. The estimated counterpoise-corrected interaction energy between H bonded SWCNT-COOH and HCOOH was about −21 kcal/mol. Such a significant interaction supports the presence of a strong interaction between “interaction hot spots” of nanotubes with a polymeric SP molecule, which leads to better mixing with a polar environment (water). The obtained theoretical results could improve our understanding about the formation of well-dispersed nanocarbon material in a superplasticizer leading to easier introduction of SWCNTs (and MWCNTs) to cement paste and concrete mix.

As result of the conducted biological studies, no clear effect of the cement substrate with the addition of MWCNTs on the studied moss species’ vitality was observed. In the case of *Polytrichum formosum*, composite modification did not induce a statistically significant effect. However, notable vitality changes of *Pseudoscleropodium purum* were observed. It should be concluded that the effect of the cement paste with the addition of MWCNTs is not clear-cut and will depend on the adaptability of the individual moss species. However, the addition of nanoparticles did not have a negative effect on the functioning of the species *Polytrichum formosum* and *Pseudoscleropodium purum*. Moreover, higher *Pseudoscleropodium purum* PSII activity on MWCNT-cement was observed compared to the control substrate. The obtained results confirmed the hypothesis that mosses could be used as bioindicators of nanomodified building materials’ environmental effect. Cement paste with MWCNTs does not have a destructive effect on the studied mosses’ viability.

## Figures and Tables

**Table 1:** Position (in cm^−1^) and tentative assignment of the FT-IR absorption bands ^a^ of the superplasticizer.

Peak	Wavenumber	Assignment	Structural Fragment
1	3462	OH str	-OH hydroxyl group
2	2944	CH_2_(O) asym str	-CH_2_(O) methylene group adjacent to oxygen
3	2888	CH_2_(O) sym str	-CH_2_(O) methylene group adjacent to oxygen
4	~2862	CH_2_ str	-CH_2_- methylene group in polymer chain
5	~2806	CH str	Methine group in polymer chain (near C=O)
6	2741	2× CH_2_ sciss	Overtone of the 1360cm^−1^ band
7	2692	2× CH_2_ sciss	Overtone of the 1343cm^−1^ band
8	1728	C=O str	O=C-OR ester
9	1705	C=O str	O=C-OH carboxyl (acid)
10	1578	COO^−^ asym str	-COO^−^ carboxylate (salt)
11, 12	1467, 1455	CH_2_, CH def	CH_2_ and CH aliphatic methylene and methine
13	1412	COO^−^ sym str	-COO^−^ carboxylate (salt)
14, 15	1360, 1343	CH_2_ sciss	-CH_2_- methylene group in ethylene fragment
16, 17	1280, 1242	CCO + C-O-C str	O=C-OR ester
18, 19	1149, 1110	C-O-C asym str	H_2_C-O-CH_2_- ether
20	1061	C-O (H/C) str	C-O(H) C-O(C) bond in alcohols and esters
21, 22	964, 948	CH_2_ rock	-CH_2_CH_2_- ethylene fragment
23	843	CCO bend	-CH_2_CH_2_-O- oxyethylene fragment

^a^ Mode abbreviations: str (stretch), bend (bending), def (deformation), sciss (scissoring) (in-plane bending), sym (symmetric), asym (asymmetric), rock (rocking).

**Table 2:** Vibrational bands ^a^ (in cm^−1^) in the Raman spectra of neat superplasticizer (sample A) and with addition of 3% MWCNTs (sample B).

No	A		B		Assignment
	Position	Intensity	Position	Intensity	
1			3212	vw	2G
2			2990	m	D + G
3	2937	sh			CH_2_(O) asym str
4	2906	sh			
5	2884	vs			CH_2_(O) sym str
6	2851	sh			CH_2_ str
7	2731	w			
8	2693	w			2 × CH_2_ sciss
9			2684	s	G’ (2D)
10			~2441	vw	G*
11			1620	sh	G2
12			1583	vs	G (Tangential)
13	1480	m			CH_2_, CH def
14	1397	vw			
15			1346	vs	D (Disorder)
16	1280	m			CCO + C-O-C str
17	1233	vw			CCO + C-O-C str
18			~1220	vw	
19	1142	m			C-O-C asym str
20	1064	w			C-O(H/C) str
21	843	m			CCO bend
22	579	vw			
23	534	vw			
24	360	vw			
25	276	w			

^a^ Mode abbreviations: str (stretch); bend (bending); def (deformation), sciss (scissoring) (in-plane bending); sym (symmetric), asym (asymmetric); m (middle); w (weak); vs (very strong); vw (very weak) and sh (shoulder).

**Table 3:** Raw and CP-corrected interaction energy (in kcal/mol) of formic and acetic acid dimers. The magnitude of dispersion and BSSE are also evaluated.

Method	Interaction Energy	Dispersion	% Dispersion	BSSE
Formic acid dimer				
Optimized and SP calculated with 6-31+G*				
BLYP (raw)	−17.23			1.19
BLYP (CP)	−16.04			
Optimized with 6-31+G*, SP calculated with aug-cc-pVTZ				
BLYP (raw)	−17.41			0.23
BLYP (CP)	−17.18			
Optimized with 6-31+G*, SP calculated with aug-cc-pV5Z				
BLYP (raw)	−17.34			0.02
BLYP (CP)	−17.32			
Optimized and SP calculated with aug-cc-pVTZ				
BLYP (raw)	−17.53			0.25
BLYP (CP)	−17.28			
BLYPD3BJ (raw)	−20.89	−3.36	16.08	0.26
BLYPD3BJ (CP)	−20.63	−3.35	16.24	
B3LYP (raw)	−18.42			0.26
B3LYP (CP)	−18.16			
B3LYPD3BJ (raw)	−21.15	−2.73	12.91	0.26
B3LYPD3BJ (CP)	−20.89		13.07	
CCSD(T)/aTZ ^a^	−19.7			
CCSD(T)/CBS ^b^	−18.61			
Acetic acid dimer				
Optimized and SP calculated with aug-cc-pVTZ				
BLYP (raw)	−18.36			0.29
BLYP (CP)	−18.07			
BLYPD3BJ (raw)	−22.00	−3.64	16.55	0.30
BLYPD3BJ (CP)	−21.70	−3.63	16.73	
B3LYP (raw)	−19.20			0.29
B3LYP (CP)	−18.91			
B3LYPD3BJ (raw)	−22.16	−2.96	13.36	0.29
B3LYPD3BJ (CP)	−21.87		13.53	
CCSD(T)/aTZ ^a^	−20.8			
Lit. ^b^	−14.16			

^a^ Single-point CCSD(T) on MP2/aug-cc-pVTZ geometry, from ref. [104]; ^b^ CCSD(T)/CBS(aTZ,aQZ) result, from ref. [111].

**Table 4:** Basic statistical parameters of photosynthetic activity values of bryophytes.

Species	Sample	Mean	Median	Min–Max	Geom. Mean	Harmonic Mean	SD	Variance	Range	Skewness	Kurtosis	Sum
*Pf*	Control	0.614	0.670	0.147–0.747	0.590	0.552	0.142	0.020	0.600	−1.60	1.88	49.7
	Cement	0.695	0.724	0.218–0.796	0.687	0.673	0.087	0.008	0.578	−3.43	13.7	56.3
	MWCNT-Cement	0.696	0.721	0.358–0.761	0.691	0.685	0.074	0.005	0.403	−2.38	5.97	56.4
*Pp*	Control	0.602	0.664	0.142–0.740	0.564	0.499	0.162	0.026	0.598	−1.81	2.44	48.7
	Cement	0.471	0.492	0.135–0.746	0.417	0.353	0.200	0.040	0.611	−0.365	−1.20	38.2
	MWCNT-Cement	0.554	0.621	0.130–0.766	0.510	0.448	0.182	0.033	0.636	−1.00	−0.181	44.9

**Table 5:** Statistically significant differences between substrate types and their effect on PSII values (non-parametric Mann–Whitney U test) ^a^.

Data	Sum.Rang	Sum.Rang	U	Z	*p*	Z Correct.	*p*
	*Pf*						
Control vs. cement	**4895**	**8308**	**1574**	**−5.71**	***	**−5.72**	***
Control vs. MWCNT-cement	**4944**	**8259**	**1623**	**−5.55**	***	**−5.55**	***
Cement vs. MWCNT-cement	6643	6560	3239	0.137	0.891	0.137	0.891
	*Pp*						
Control vs. cement	**7918**	**5285**	**1964**	**4.41**	***	**4.41**	***
Control vs. MWCNT-cement	7100	6104	2783	1.67	0.096	1.67	0.096
Cement vs. MWCNT-cement	**5769**	**7434**	**2448**	**−2.79**	**	**−2.79**	**
	*Pf* vs. *Pp*						
Control vs. cement	6729	6474	3153	0.425	0.671	0.425	0.671
Control vs. MWCNT-cement	**9035**	**4168**	**847**	**8.15**	***	**8.15**	***
Cement vs. MWCNT-cement	**8588**	**4615**	**1294**	**6.65**	***	**6.65**	***

^a^ Bold values indicate statistical significance at the level of: ** *p* < 0.01, *** *p* < 0.001.

**Table 6:** Compositions (in g) of studied cement probes.

Composite	CEM I	Water	MWCNT-SP
Cement	250	300	-
MWCNT-Cement	250	300	2.75

## Data Availability

Data are contained within the article and Supplementary Materials.

## Supplementary Materials

The following supporting information can be downloaded at: https://www.mdpi.com/article/10.3390/molecules29225379/s1, Figure S1: FTIR spectra of MWCNT; Figure S2: BLYPD3BJ and B3LYPD3BJ (in parenthesis) optimized structures of linear water dimer (the aug-cc-pVTZ basis set was used, distances are in Å, angles in degrees and interaction energy in kcal/mol); Figure S3: BLYPD3BJ and BLYP (in parenthesis) optimized structures of (A) *trans*-formic and (B) *trans*-acetic acid monomers (the aug-cc-pVTZ basis set was used, distances are in Å); Figure S4: BLYPD3BJ/aVTZ and BLYP/aVTZ optimized structures of acetic acid dimer (interatomic distances in Å); Figure S5: The predicted Raman spectrum (20 cm^−1^ linewidth is used) of optimized BLYPD3BJ/6-311++G** structure of model fragment of zigzag (5,0) SWCNT-COOH composed from five “belts”; Figure S6: Chemical formula of studied superplasticizer; Figure S7: Photos of MWCN-SP (A) before and (B) after mixing; Figure S8: Moss cultivation in phytotron rack on three types of substrate: control, cement composite and MWCNT-modified cement composite; Table S1: DFT predicted (A) structural and vibrational parameters of water monomer using aug-cc-pVTZ basis set and their (B) deviations from experiment. Experimental and benchmark theoretical values are given for comparison (bonds in Å, angle in degrees and wavenumbers in cm^−1^); Table S2: DFT calculated structural parameters, interaction energy and vibration frequencies of water dimer are compared with experiment and benchmark calculations (distances in Å, angles in degrees, wavenumbers in cm^−1^ and interaction energy in kcal/mol); Table S3: (A) BLYPD3BJ, BLYP, B3LYPD3BJ and B3LYP optimized bond lengths (in Å) of *trans*-formic and *trans*-acetic acid monomers and (B) the corresponding deviations from experiment. The aug-cc-pVTZ basis set was used. Experimental and benchmark parameters are included for comparison; Table S4: (A) Selected experimental and theoretical benchmark wavenumbers of formic acid monomer, (B) BLYP and BLYPD3BJ and (C) B3LYP and B3LYPD3BJ calculated harmonic and anharmonic values using the aug-cc-pVTZ basis set; Table S5: BLYP, BLYPD3BJ, B3LYP and B3LYPD3BJ calculated harmonic and anharmonic frequencies of acetic acid monomer using the aVTZ basis set; Table S6: BLYP, BLYPD3BJ, B3LYP and B3LYPD3BJ predicted formic acid dimer interatomic distances using aVTZ basis set and their deviations from experiment. Experimental and benchmark parameters are included for comparison; Table S7: Experimental and benchmark frequencies of formic acid dimer; Table S8: BLYP, BLYPD3BJ, B3LYP and B3LYPD3BJ calculated harmonic and anharmonic frequencies of formic acid dimer. aVTZ basis set is used and RMS deviations from experiment are also shown; Table S9: BLYP/aVTZ, BLYPD3BJ/aVTZ, B3LYP/aVTZ and B3LYPD3BJ/aVTZ calculated harmonic and anharmonic frequencies of acetic acid dimer; Table S10: Raw and CP-corrected interaction energy (in kcal/mol) of formic and acetic acid dimers. The magnitude of dispersion and BSSE is also evaluated, see refs. [144,145].
