# Supplementary material for: Carbon Nanotubes in Cement—A New Approach for Building Composites and Its Influence on Environmental Effect of Material
Source: Molecules. 2024 Nov 14;29(22):5379. doi: 10.3390/molecules29225379 (PMC11597654; doi:10.3390/molecules29225379)
Supplement: Supplementary file 1 [file molecules-29-05379-s001.zip › molecules-3306275-supplementary.pdf]

SUPLEMENTARY MATERIALS

For

Carbon Nanotubes in Cement—A New Approach for Building Composites and Its Influence on Environmental Effect of Material

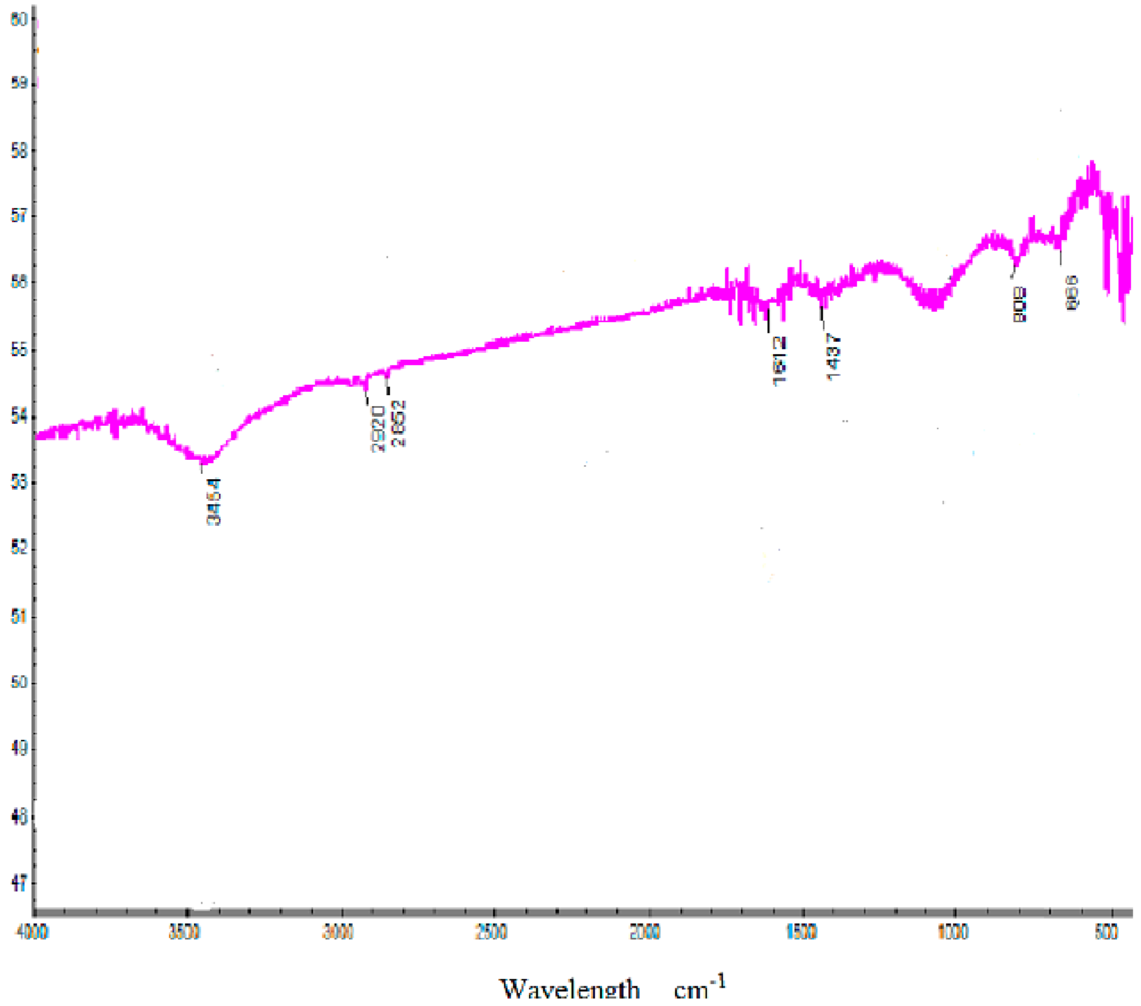

Figure S1. FTIR spectra of MWCNT.

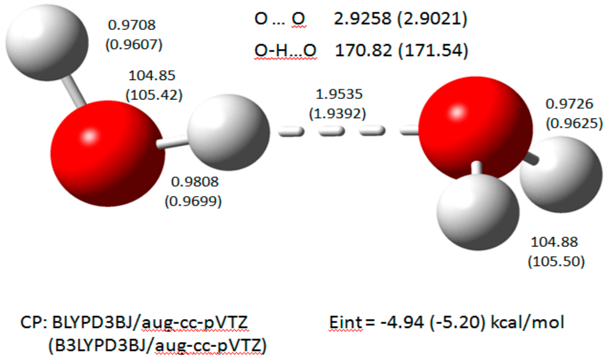

Figure S2. BLYPD3BJ and B3LYPD3BJ (in parenthesis) optimized structures of linear water dimer (the aug-cc-pVTZ basis set was used, distances are in Å, angles in degrees and interaction energy in kcal/mol).

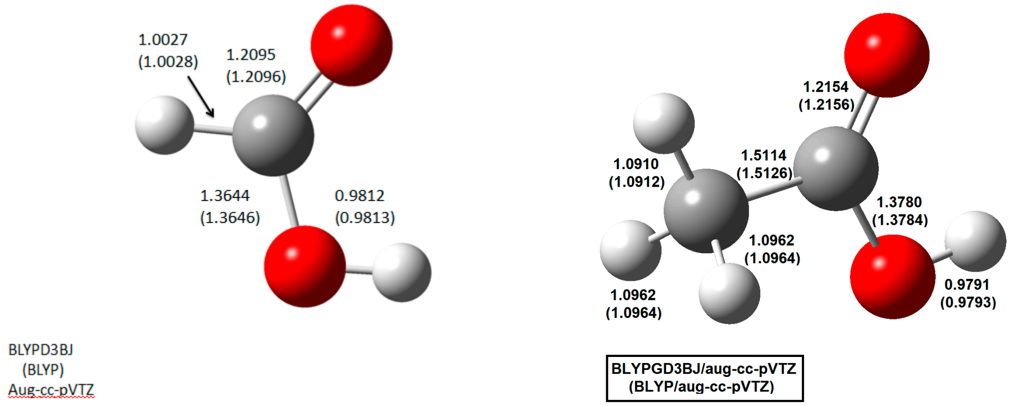

Figure S3. BLYPD3BJ and BLYP (in parenthesis) optimized structures of (A) *trans*-formic and (B) *trans*-acetic acid monomers (the aug-cc-pVTZ basis set was used, distances are in Å).

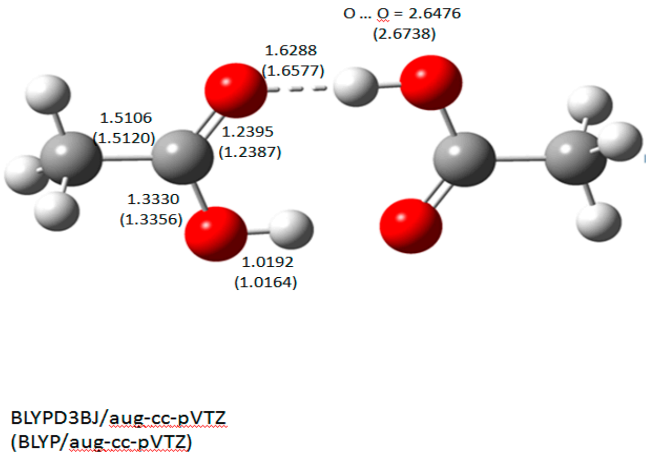

Figure S4. BLYPD3BJ/aVTZ and BLYP/aVTZ optimized structures of acetic acid dimer (interatomic distances in Å).

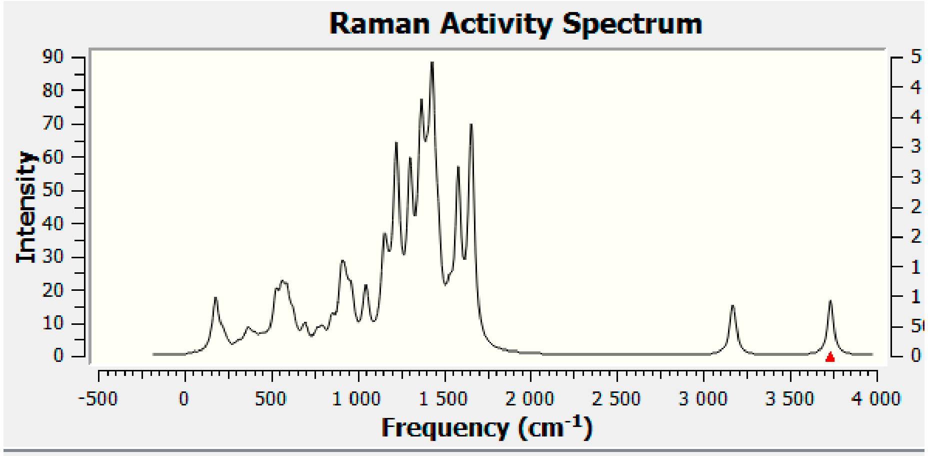

Figure S5. The predicted Raman spectrum (20 cm^{-1} linewidth is used) of optimized BLYPD3BJ/6-311++G** structure of model fragment of zigzag (5,0) SWCNT-COOH composed from five "belts"

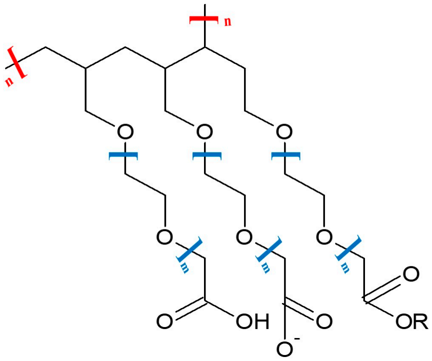

Figure S6. Chemical formula of studied superplasticizer

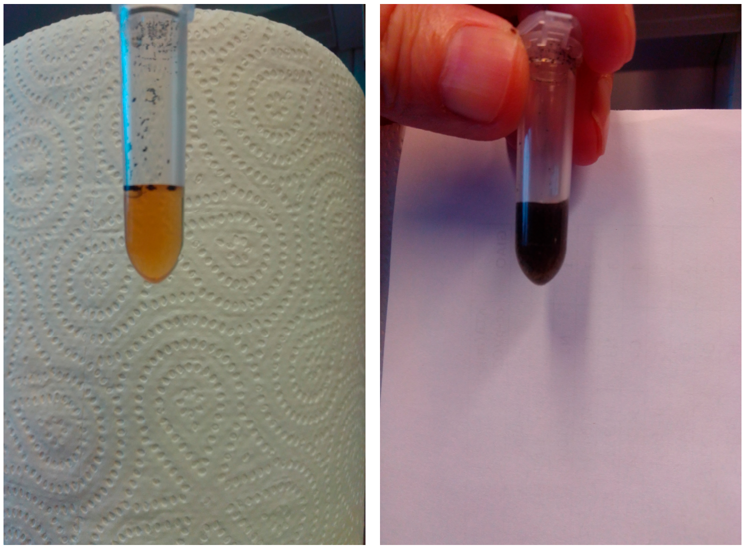

A B

Figure S7. Photos of MWCN-SP (A) before and (B) after mixing

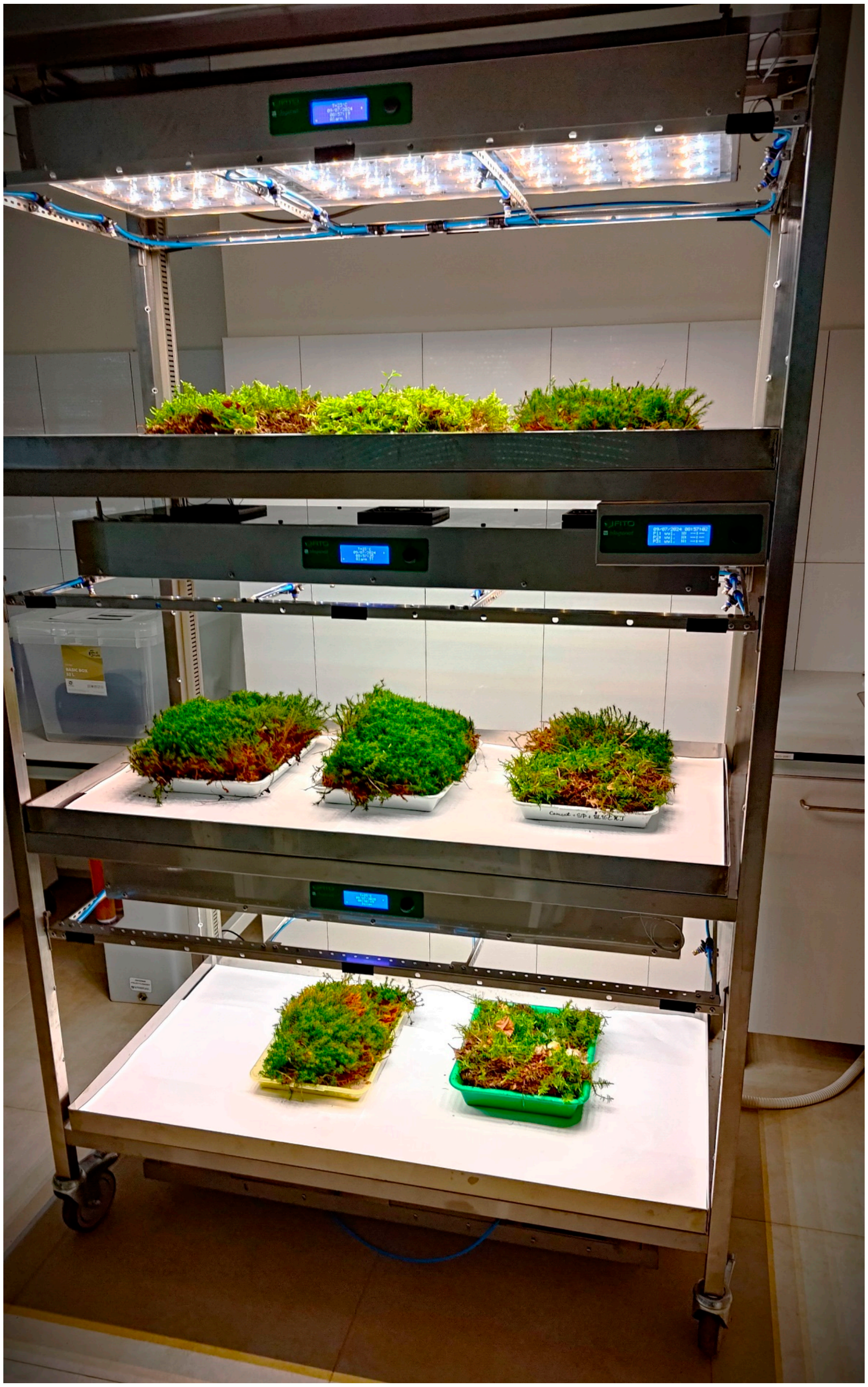

Figure S8. Moss cultivation in phytotron rack on three types of substrate: control, cement composite and MWCNT-modified cement composite

Table S1 DFT predicted (A) structural and vibrational parameters of water monomer using aug-cc-pVTZ basis set and their (B) deviations from experiment. Experimental and benchmark theoretical values are given for comparison (bonds in Å, angle in degrees and wavenumbers in cm^{-1}).

(A)

Parameter	**Benedict[a]**	CCSD(T)[b]	B3LYP	B3LYPD3BJ	BLYP	BLYPD3BJ
	Geometry					
OH[a]	**0.9572**	0.9583	0.9618	0.9618	0.9719	0.9719
HOH[a]	**104.52**	104.45	105.09	105.08	104.48	104.47
Harmonic[a]	Harmonic[a]					
ω1	**3832.2**	3834.78	3796.51	3796.45	3654.88	3654.76
ω2	**1648.5**	1649.57	1627.32	1627.36	1595.93	1595.98
ω3	**3942.5**	3946.31	3899.17	3899.07	3756.41	3756.24
Fundamental[a]	Anarmonic treated as fundamental					
γ1	**3656.6**	3656.87	3628.04	3627.78	3480.58	3480.13
γ2	**1594.6**	1594.98	1574.65	1574.69	1543.92	1543.96
γ3	**3755.93**	3756.09	3716.94	3716.64	3567.97	3567.48
Fundamental[a]	Harmonic treated as fundamental					
γ1	**3656.6**	3834.78	3796.51	3796.45	3654.88	3654.76
γ2	**1594.6**	1649.57	1627.32	1627.36	1595.93	1595.98
γ3	**3755.93**	3946.31	3899.17	3899.07	3756.41	3756.24

(B)

Deviation from experiment					
Parameter	CCSD(T)	B3LYP	B3LYPD3BJ	BLYP	BLYPD3BJ
	Geoometry				
OH[a]	0.0011	0.0046	0.0046	0.0147	0.0147
HOH[a]	-0.07	0.57	0.56	-0.04	-0.05
Harmonic[a]	Harmonic				
ω1	2.58	-35.69	-35.75	-177.32	-177.44
ω2	1.07	-21.18	-21.14	-52.57	-52.52
ω3	3.81	-43.33	-43.43	-186.09	-186.26
**RMS**	**2.7**	**34.6**	**34.7**	**151.5**	**151.6**
Fundamental[a]	Anharmonic treated as fundamental				
γ1	0.27	-28.56	-28.81	-176.02	-176.47
γ2	0.38	-19.95	-19.91	-50.68	-50.64
γ3	0.16	-38.99	-39.29	-187.96	-188.45
**RMS**	**0.3**	**30.2**	**30.4**	**151.5**	**151.9**
Fundamental[a]	Harmonic treated as fundamental				
γ1	178.18	139.91	139.85	-1.72	-1.84
γ2	54.97	32.72	32.76	1.33	1.38
γ3	190.38	143.24	143.14	0.48	0.31
**RMS**	**153.9**	**117.1**	**117.1**	**1.3**	**1.3**

a) Monomer harmonic and fundamentals from ref. [1]: Benedict WC, Gailar N, Plyler EK (1956) Rotation-vibration spectra of deuterated water vapor. J Chem Phys 24:1139–1165;
b) from ref. [2] David Feller, Kirk A. Peterson, High level coupled cluster determination of the structure, frequencies, and heat of formation of water, J. Chem. Phys. 131 (2009) 154306. CCSD(T)/CBS(67) results with additional corrections to vibrations.

Table S2 DFT calculated structural parameters, interaction energy and vibration frequencies of water dimer are compared with experiment and benchmark calculations (distances in Å, angles in degrees, wavenumbers in cm^{-1} and interaction energy in kcal/mol).

Parameter	Lane[a] "Best"	CCSD(T) [b]	B3LYPD3BJ	BLYPD3BJ	Deviation from Exp. or CC result		
	CCSDT	ha5Z[b]	aTZ		CCSD(T)	B3LYPD3BJ	BLYPD3BJ
OHd1	0.9569	0.9576	0.9607	0.9708	0.0005	0.0038	0.0139
OHd	0.9641	0.9647	0.9699	0.9808	0.0005	0.0058	0.0167
OHa	0.9584	0.9591	0.9625	0.9726	0.0006	0.0041	0.0142
HOHd	104.85	104.76	105.42	104.85	-0.05	0.57	0
HOHa	104.95	104.84	105.50	104.89	-0.06	0.55	-0.06
O-H..O	174.31	171.69	171.54	170.86	-0.01	-2.77	-3.45
O-H…O		1.9548	1.9392	1.9535	-	-	-
O … O	2.9092	2.9128	2.9021	2.9258	0.0036	-0.0071	0.0166
**Eint**	**-5.02**	**-4.98**	**-5.20(CP)**	**-4.94(CP)**	**0.04**	**-0.18**	**0.08**
Harmonic	Exp.[c]	ha5Z[b]					
1	88	125	131.75	131.67	37	43.75	43.67
2	103	142	153.22	211.79	39	50.22	108.79
3	108	148	161.03	214.77	40	53.03	106.77
4	150	185	191.44	333.55	35	41.44	183.55
5	310	351	370.54	403.03	41	60.54	93.03
6	520	614	635.90	819.54	94	115.9	299.54
7	1601	1650	1627.84	1679.3	49	26.84	78.3
8	1619	1670	1647.48	1709.97	51	28.48	90.97
9	3480	3754	3669.91	3497.79	274	189.91	17.79
10	3627	3830	3791.03	3525.71	203	164.03	-101.29
11	3699	3917	3870.44	3651.23	218	171.44	-47.77
12	3715	3936	3889.89	3651.27	221	174.89	-63.73
**RMS**					**161.2**	**129.3**	**144.7**
Fundamental	Exp.[c]	VPT2, haQZ[b]	VPT2	VPT2			
1	88	79	68.911	51.16	-9	-19.09	-36.84
2	103	111	132.434	128.93	8	29.43	25.93
3	108	103	151.928	120.76	-5	43.93	12.76
4	150	143	155.268	135.60	-7	5.27	-14.40
5	310	293	322.341	306.63	-17	12.34	-3.37
6	520	495	519.252	514.00	-25	-0.75	-6.00
7	1601	1599	1579.72	1549.42	-2	-21.28	-51.58
8	1619	1616	1595.33	1561.63	-3	-23.67	-57.37
9	3480	3605	3532.195	3372.09	125	52.20	-107.91
10	3627	3650	3621.271	3474.67	23	-5.73	-152.33
11	3699	3727	3695.474	3543.31	28	-3.53	-155.69
12	3715	3747	3708.977	3561.00	32	-6.02	-154.00
**RMS**					**39.90**	**24.43**	**87.18**

a) The "best" structure calculated at CCSD(T)/CBS level of theory, ref. [3] Lane JR (2013) CCSDTQ optimized geometry of water dimer. J Chem Theory Comput 9 (1):316-323. doi:10.1021/ct300832f; b) ref. [4] Howard JC, Gray JL, Hardwick AJ, Nguyen LT, Tschumper GS (2014) Getting down to the fundamentals of hydrogen bonding: Anharmonic vibrational frequencies of (HF)2 and (H2O)2 from Ab initio electronic structure computations. J. Chem. Theory Comput. 10 (12):5426-5435; c) water dimer, ref. [5] Dyke TR, Mack KM, Muenter JS (1976) The structure of water dimer from molecular beam electric resonance spectroscopy. J Chem Phys 71 (2):498-510; ref. [6] Yeo GA, Ford TA (1992) The combined use of ab initio molecular orbital theory and matrix isolation infrared spectroscopy in the study of molecular interactions. Struct Chem 3 (2):75-93; Ref. [7] Wuelfert S, Herren D, Leutwyler S (1987) Supersonic jet CARS spectra of small water clusters. J Chem Phys 86 (6):3751-3753; ref. [8] Amos RD (1986) Structures, harmonic frequencies and infrared intensities of the dimers of H2O and H2S. Chem Phys 104 (1):145-151; ref.[9] Bentwood RM, Barnes AJ, Orville-Thomas WJ (1980) Studies of intermolecular interactions by matrix isolation vibrational spectroscopy: Self-association of water. J Mol Spectrosc 84 (2):391-404.

Table S3 (A) BLYPD3BJ, BLYP, B3LYPD3BJ and B3LYP optimized bond lengths (in Å) of *trans*-formic and *trans*-acetic acid monomers and (B) the corresponding deviations from experiment. The aug-cc-pVTZ basis set was used. Experimental and benchmark parameters are included for comparison.

S3 (A)

Parameter	Exp. [a]	CCSD(T) / aVQZ[b]	BLYP	BLYPD3BJ	B3LYP	B3LYPD3BJ
Formic acid						
C=O	1.202	1.2004	1.2096	1.2095	1.1977	1.1977
C-O	1.343	1.3442	1.3646	1.3644	1.3451	1.3449
O-H	0.972	0.9675	0.9813	0.9812	0.9703	0.9703
C-H	1.097	1.0940	1.1028	1.1027	1.0960	1.0959
Acetic Acid[c]						
C=O	1.214[c]	1.2051[d]	1.2156	1.2154	1.2036	1.2035
C-O	1.364[c]	1.3373 [d]	1.3784	1.3780	1.3574	1.3571
C-C	1.520[c]	1.4920 [d]	1.5126	1.5114	1.5018	1.5009
O-H	0.970[c]	0.9608 [d]	0.9793	0.9791	0.9686	0.9685
C-H	1.102[c]	1.0856 [d]	1.0947	1.0964	1.0883	1.0881

S3 (B)

Parameter	Exp. [a]	Deviation from Experiment				
		CCSD(T) /aVQZ[b]	BLYP	BLYPD3BJ	B3LYP	B3LYPD3BJ
Formic acid						
C=O	1.202	-0.0016	0.0076	0.0075	-0.0043	-0.0043
C-O	1.343	0.0012	0.0216	0.0214	0.0021	0.0019
O-H	0.972	-0.0045	0.0093	0.0092	-0.0017	-0.0017
C-H	1.097	-0.0030	0.0058	0.0057	-0.0010	-0.0011
Acetic Acid						
C=O	1.214[c]	-0.0089[d]	0.0016	0.0014	-0.0104	-0.0105
C-O	1.364[c]	-0.0267[d]	0.0144	0.0140	-0.0093	-0.0069
C-C	1.520[c]	-0.0280[d]	-0.0074	-0.0086	-0.0182	-0.0191
O-H	0.970[c]	-0.0092[d]	0.0093	0.0091	-0.0014	-0.0015
C-H	1.102[c]	-0.0164[d]	-0.0073	-0.0056	-0.0137	-0.0139

[a]NIST[10]: Herzberg, G., Electronic spectra and electronic structure of polyatomic molecules, Van Nostrand, New York, 1966; [b]from Xantheas, fc CCSD(T)/aVQZ , ref [11] [12]; [c]experimental results for acetic acid [13-16] [d]NIST, ae CCSD/aug-cc-pVTZ result[17]

Table S4 (A) Selected experimental and theoretical benchmark wavenumbers of formic acid monomer, (B) BLYP and BLYPD3BJ and (C) B3LYP and B3LYPD3BJ calculated harmonic and anharmonic values using the aug-cc-pVTZ basis set.

S4 A

No	Exp.			Theory		
	Gas, Raman[a]	IR, Marechal[b]	Fausto, Xantheas [c]	Luber[d]	Xantheas[e]	Tew [f]
1	624.9	612	628	626.17	630	633
2	642.0	640	639	640.73	671	672
3	1033.	…	1037	1033.47	1055	1057
4	1103.8	1104	1101	1104.85	1135	1140
5	1223	1218	1306	1306.2	1314	1319
6	1380.6	…	1384	1379.05	1408	1410
7	1776.6	1775	1844	1776.83	1810	1818
8	2942.0	2941	2956	2942.06	3092	3093
9	3568.9	3567	3554	3570.5	3761	3766

[a] Gas, Raman, ref. [18]; [b] IR gas, from ref. [19]; [c] Exp. Fausto, Xantheas, from ref. [16] [12]; [d] from ref. [20], Luber; [e] Xantheas, harmonic CCSD(T)/aVQZ, from ref. [12]; [f]CCSD(T)(F12*)/cc-pVTZ-F12*, from ref. [21]

S4 B BLYP and BLYPD3BJ calculated harmonic and anharmonic frequencies of formic acid monomer using the aug-cc-pVTZ basis set (this work).

No	BLYP	BLYPD3BJ	BLYP	BLYPD3BJ
	Harmonic		VPT2	
1	600.74	601.22	593.28	593.76
2	664.90	664.04	632.43	631.43
3	1001.19	1000.77	980.68	980.27
4	1051.44	1051.31	1017.55	1017.41
5	1249.73	1250.31	1181.69	1181.48
6	1357.63	1357.83	1330.25	1330.24
7	1728.83	1729.81	1695.4	1696.39
8	2959.73	2961.04	2796.01	2796.48
9	3558.62	3560.67	3370.31	3371.3
**RMS**				
**vs. gas (Raman)[a]**	**31.28**	**31.13**	**95.95**	**95.59**

[a] from ref. [20]

S4 C B3LYP and B3LYPD3BJ calculated harmonic and anharmonic frequencies of formic acid monomer using the aug-cc-pVTZ basis set (this work).

No	B3LYP	B3LYPD3BJ	B3LYP	B3LYPD3BJ
	Harmonic		VPT2	
1	628.58	629.07	622.59	623.09
2	674.49	674.67	641.28	641.48
3	1051.69	1051.55	1031.80	1031.65
4	1121.22	1121.66	1088.99	1089.46
5	1297.97	1298.72	1215.55	1294.26
6	1401.72	1401.91	1373.76	1373.96
7	1811.17	1811.40	1778.57	1778.81
8	3048.13	3048.70	2893.19	2893.07
9	3716.22	3716.51	3528.40	3528.12
**RMS**				
**vs gas (Raman)[a]**	**68.27**	**68.57**	**22.00**	**32.31**

[a] from ref. [20]

Table S5 BLYP, BLYPD3BJ, B3LYP and B3LYPD3BJ calculated harmonic and anharmonic frequencies of acetic acid monomer [22] using the aVTZ basis set.

No	Exp.[a]	BLYP		BLYPD3BJ		B3LYP		B3LYPD3BJ	
		Harmonic	Anharmonic	Harmonic	Anharmonic	Harmonic	Anharmonic	Harmonic	Anharmonic
1	103	-	-	-	-	71.23	5.75	71.68	7.36
2	423	411.67	409.97	412.50	410.78	424.13	423.57	424.75	424.20
3	533.5	529.50	517.57	528.60	516.68	545.89	536.10	545.17	535.25
4	577.5	559.74	550.59	560.56	551.37	584.24	576.39	584.83	576.96
5	635	644.26	628.62	644.07	628.48	663.10	649.84	662.93	649.77
6	844	814.62	794.15	817.13	796.59	857.53	839.47	859.42	841.34
7	986	957.11	934.65	958.16	935.72	998.71	978.91	999.39	979.29
8	1047	1036.21	1012.20	1036.71	1012.80	1070.25	1045.95	1070.62	1046.36
9	1150	1146.95	1107.50	1148.13	1108.60	1200.99	1159.22	1201.76	1159.74
10	1325	1283.69	1207.35	1285.22	1208.29	1334.70	1326.87	1335.78	1327.30
11	1381	1363.58	1327.50	1363.82	1328.34	1408.45	1369.16	1408.82	1369.76
12	1436	1437.68	1394.45	1437.90	1394.71	1471.97	1433.11	1472.14	1433.34
13	1441	1442.33	1387.85	1442.54	1388.14	1478.08	1429.51	1478.24	1429.96
14	1780.5	1729.32	1694.74	1730.84	1696.35	1811.98	1779.80	1813.11	1780.99
15	2953	2980.06	2860.96	2981.44	2861.05	3052.42	2942.06	3053.52	2942.33
16	3007	3029.89	2885.60	3031.57	2886.05	3108.64	2974.21	3109.97	2974.97
17	3057	3082.12	2930.02	3083.90	2930.82	3159.57	3010.45	3160.99	3011.35
18	3568	3584.65	3399.91	3586.11	3400.31	3737.61	3560.12	3738.89	3560.61
RMS	-								

[a] In solid argon, from ref. [22]

Table S6 BLYP, BLYPD3BJ, B3LYP and B3LYPD3BJ predicted formic acid dimer interatomic distances using aVTZ basis set and their deviations from experiment. Experimental[a] and benchmark[b] parameters are included for comparison.

	Exp.[a]	CCSD(T)[b]	BLYP	BLYPD3BJ	Deviation	
		CBS(aT,aQ)			BLYP	BLYPD3BJ
C=O	1.220	1.215	1.2320	1.2328	0.012	0.0128
C-O	1.323	1.307	1.3256	1.3234	0.0026	0.0004
O-H	1.036	0.994	1.0172	1.0199	-0.0188	-0.0161
C-H	1.079	1.092	1.1014	1.1012	0.0224	0.0222
O…H	-	1.668	1.6672	1.6396	-	-
O…O	2.696	2.663	2.6843	2.6595	-0.0117	-0.0365
					Deviation	
		CBS(aT,aQ) [b]	B3LYP	B3LYPD3BJ	B3LYP	B3LYPD3BJ
C=O	1.220	1.215	1.2184	1.2190	-0.0016	-0.0010
C-O	1.323	1.307	1.3099	1.3085	-0.0131	-0.0145
O-H	1.036	0.994	1.0024	1.0042	-0.0336	-0.0318
C-H	1.079	1.092	1.0947	1.0946	0.0157	0.0156
O…H	-	1.668	1.6650	1.6441	-	-
O…O	2.680	2.663	2.6672	2.6482	-0.0288	-0.0478

[a]from ref. [23]; [b]from ref. [24]

Table S7 Experimental and benchmark frequencies of formic acid dimer.

No	Exp.[a] Pettersson	Exp [b] Sotiris	CCSD(T)/aQZ[c] Bowman
1	68	69	72
2	165	161	167
3	163	168	177
4	194	194	209
5	251	242	255
6	248	264	275
7	680	677	684
8	697	698	713
9	947	911	963
10		922	986
11	1050	1050	1079
12	1062	1060	1099
13	1214	1214	1252
14	1218	1218	1256
15	1365	1364	1405
16	1375	1375	1409
17	1415	1415	1455
18	1445	1454	1481
19	1660	1670	1713
20	1728	1746	1777
21	2802	2900	3099
22	2944	2939	3103
23	2949	2949	3204
24	3000	3084	3306

[a]From ref. [18, 25]; [b]From ref. [12]; [c]From ref. [12, 26]

Table S8 BLYP, BLYPD3BJ, B3LYP and B3LYPD3BJ calculated harmonic and anharmonic frequencies of formic acid dimer. aVTZ basis set is used and RMS deviations from experiment are also shown.

No	BLYP		BLYPD3BJ		B3LYP		B3LYPD3BJ	
	Harmonic	Anharmonic	Harmonic	Anharmonic	Harmonic	Anharmonic	Harmonic	Anharmonic
1	75.254	48.362	72.578	45.314	76.778	59.307	74.599	67.731
2	173.504	161.595	177.495	166.838	175.021	165.740	177.693	170.450
3	183.803	174.545	183.432	173.519	185.638	182.096	185.202	182.984
4	206.687	192.847	218.114	204.002	211.761	199.170	221.103	207.620
5	256.775	236.073	257.259	244.352	260.373	246.334	260.821	252.497
6	277.990	270.245	294.608	289.630	281.168	273.208	294.060	287.695
7	662.333	655.046	665.108	657.749	688.968	682.755	691.042	684.630
8	698.500	692.209	703.136	697.929	722.653	716.343	726.102	720.727
9	962.483	881.082	973.798	908.021	982.117	926.366	992.389	936.618
10	968.177	919.447	975.09	953.513	1002.119	949.867	1009.442	959.891
11	1033.979	1022.866	1037.638	1033.008	1078.634	1063.084	1079.916	1066.222
12	1067.695	1049.825	1075.818	1030.153	1101.272	1087.361	1105.499	1092.562
13	1192.391	1165.525	1200.363	1175.497	1256.150	1229.536	1261.832	1236.582
14	1197.847	1169.502	1205.474	1178.570	1259.485	1227.808	1264.930	1244.108
15	1354.373	1307.113	1356.935	1318.583	1401.633	1364.762	1403.867	1367.263
16	1355.183	1316.441	1357.132	1313.240	1403.746	1367.543	1405.162	1369.238
17	1411.596	1371.992	1418.078	1380.442	1448.830	1405.954	1453.612	1411.060
18	1435.641	1383.286	1443.552	1385.709	1478.228	1425.781	1484.902	1432.508
19	1607.869	1539.891	1603.166	1537.179	1692.016	1646.654	1688.332	1619.391
20	1680.448	1637.959	1680.329	1636.967	1766.242	1726.006	1766.222	1726.258
21	2845.443	2420.982	2792.100	2292.666	3038.428	2636.879	3000.957	2592.076
22	2941.531	2602.303	2910.952	2537.044	3054.214	2887.110	3051.429	2919.249
23	2976.989	2805.642	2978.166	2804.409	3065.639	2912.631	3065.410	2907.183
24	2999.649	2813.759	2989.530	2796.201	3159.278	2861.741	3132.382	2732.167
**RMS Pettersson**	**22.48**	**125.80**	**22.82**	**151.86**	**73.41**	**48.77**	**67.22**	**73.81**
	**65.76**	**67.08**	**66.47**	**66.52**	**66.40**	**65.67**	**67.22**	**65.98**

[a]With respect to experimental data from ref. [18, 25]

Table S9 BLYP/aVTZ, BLYPD3BJ/aVTZ, B3LYP/aVTZ and B3LYPD3BJ/aVTZ calculated harmonic and anharmonic frequencies of acetic acid dimer.

No	BLYP		BLYPD3BJ		B3LYP		B3LYPD3BJ	
	Harmonic	Anharmonic	Harmonic	Anharmonic	Harmonic	Anharmonic	Harmonic	Anharmonic
1	59.25	22.44	57.50	22.60	46.90	29.03	61.51	42.20
2	75.31	52.42	72.99	58.33	62.90	41.55	75.02	73.47
3	119.43	98.89	119.43	91.43	76.96	72.49	121.48	113.43
4	160.51	137.64	164.16	142.43	121.48	113.36	164.92	164.92
5	179.31	168.93	187.79	183.35	162.62	162.39	**188.50**	**194.15**
6	179.40	172.52	189.03	177.06	181.72	180.27	191.66	177.66
7	431.62	428.09	432.85	428.73	183.42	166.85	**444.49**	**445.10**
8	474.34	471.55	482.71	480.81	**443.54**	**444.47**	**492.36**	**494.78**
9	581.64	564.99	581.57	562.84	603.06	595.09	603.00	594.21
10	589.14	572.03	589.10	570.09	611.04	601.91	611.00	600.98
11	604.20	592.32	607.93	600.03	627.82	615.54	630.72	616.61
12	615.19	610.16	618.42	613.28	637.07	634.31	639.50	636.50
13	863.26	848.61	868.23	855.02	901.81	886.51	905.49	890.85
14	866.88	852.32	871.84	857.66	905.71	890.61	909.42	895.10
15	968.60	906.44	984.78	940.91	982.64	905.65	995.46	930.90
16	998.53	979.05	1000.68	981.22	1021.46	999.25	1031.33	1015.32
17	1002.85	982.46	1005.19	984.91	1029.92	1013.84	1033.82	1007.60
18	1004.16	987.38	1019.74	983.22	1033.48	1014.93	1035.09	1017.13
19	1040.16	1021.60	1040.95	1022.73	1074.03	1056.97	1074.59	1055.67
20	1040.72	1012.08	1041.63	1017.11	1074.48	1052.91	1074.99	1051.01
21	1251.38	1212.58	1258.87	1226.12	1316.86	1264.47	1322.40	1273.42
22	1273.47	1237.21	1281.90	1243.45	1332.71	1284.68	1338.60	1287.67
23	1358.10	1296.20	1359.38	1302.33	1397.17	1339.51	1399.07	1346.82
24	1359.52	1311.02	1360.06	1312.38	1400.35	1359.93	1401.27	1362.63
25	1414.38	1381.65	1421.30	1401.54	1461.30	1412.79	1464.44	1403.39
26	1424.36	1355.26	1426.30	1355.65	1464.44	1401.43	1465.08	1412.34
27	1435.82	1393.24	1436.75	1377.71	1472.46	1446.09	1474.97	1452.78
28	1442.58	1394.30	1442.79	1394.62	1478.19	1432.00	1478.34	1432.64
29	1442.62	1393.39	1442.83	1394.81	1478.21	1434.10	1478.36	1434.77
30	1449.36	1418.39	1455.15	1425.48	1494.80	1465.67	1500.82	1464.15
31	1617.46	1569.84	1615.44	1533.74	1700.12	1656.34	1698.09	1654.36
32	1666.75	1619.52	1667.08	1616.23	1752.89	1717.09	1753.19	1716.71
33	2844.72	2379.22	2788.43	2276.74	3037.57	2683.66	2996.86	2580.45
34	2963.33	2580.16	2918.72	2529.40	3052.64	2941.40	3053.77	2937.35
35	2979.91	2859.10	2981.39	2851.96	3053.30	2931.28	3053.94	2931.75
36	2980.00	2853.69	2981.45	2850.93	3108.85	2983.14	3110.28	2965.69
37	3029.65	2882.91	3031.52	2872.06	3108.87	2953.79	3110.29	2961.33
38	3029.67	2876.13	3031.53	2871.42	3146.50	2818.52	3113.88	2754.06
39	3081.14	2923.36	3082.86	2925.87	3159.23	3009.48	3160.57	3004.78
40	3081.15	2929.70	3082.87	2927.25	3159.53	3012.46	3160.60	3010.83

Table S10 Raw and CP-corrected interaction energy (in kcal/mol) of formic and acetic acid dimers. The magnitude of dispersion and BSSE is also evaluated.

Method	Interaction energy	Dispersion	% Dispersion	BSSE
Formic acid dimer				
Optimized and SP calculated with 6-31+G*				
BLYP(raw)	-17.23			1.19
BLYP(CP)	-16.04			
Optimized with 6-31+G*, SP calculated with aug-cc-pVTZ				
BLYP(raw)	-17.41			0.23
BLYP(CP)	-17.18			
Optimized with 6-31+G*, SP calculated with aug-cc-pV5Z				
BLYP(raw)	-17.34			0.02
BLYP(CP)	-17.32			
Optimized and SP calculated with aug-cc-pVTZ				
BLYP(raw)	-17.53			0.25
BLYP(CP)	-17.28			
BLYPD3BJ(raw)	-20.89	-3.36	16.08	0.26
BLYPD3BJ(CP)	-20.63	-3.35	16.24	
B3LYP(raw)	-18.42			0.26
B3LYP(CP)	-18.16			
B3LYPD3BJ(raw)	-21.15	-2.73	12.91	
B3LYPD3BJ(CP)	-20.89		13.07	
CCSD(T)/aTZ[a]	-19.7			
CCSD(T)/CBS[b]	-18.61			
Acetic acid dimer				
Optimized and SP calculated with aug-cc-pVTZ				
BLYP(raw)	-18.36			0.29
BLYP(CP)	-18.07			
BLYPD3BJ(raw)	-22.00	-3.64	16.55	0.30
BLYPD3BJ(CP)	-21.70	-3.63	16.73	
B3LYP(raw)	-19.20			0.29
B3LYP(CP)	-18.91			
B3LYPD3BJ(raw)	-22.16	-2.96	13.36	
B3LYPD3BJ(CP)	-21.87		13.53	
CCSD(T)/aTZ[a]	-20.8			
Lit.[b]	-14.16			

[a] Single point CCSD(T) on MP2/aug-cc-pVTZ geometry, from ref. [27];

[b] CCSD(T)/CBS(aTZ,aQZ) result, from ref. [28]

References

1. Benedict, W. S.; Gailar, N.; Plyler, E. K., Rotation-Vibration Spectra of Deuterated Water Vapor. *J. Chem. Phys.* **1956,** 24, (6), 1139-1165.
2. Feller, D.; Peterson, K. A., High level coupled cluster determination of the structure, frequencies, and heat of formation of water. *J. Chem. Phys.* **2009,** 131, art. no. 154306.
3. Lane, J. R., CCSDTQ optimized geometry of water dimer. *J. Chem. Theory Comput.* **2013,** 9, (1), 316-323.
4. Howard, J. C.; Gray, J. L.; Hardwick, A. J.; Nguyen, L. T.; Tschumper, G. S., Getting down to the fundamentals of hydrogen bonding: Anharmonic vibrational frequencies of (HF)2 and (H2O)2 from Ab initio electronic structure computations. *J. Chem. Theory Comput.* **2014,** 10, (12), 5426-5435.
5. Dyke, T. R.; Mack, K. M.; Muenter, J. S., The structure of water dimer from molecular beam electric resonance spectroscopy. *J. Chem. Phys.* **1976,** 71, (2), 498-510.
6. Yeo, G. A.; Ford, T. A., The combined use of ab initio molecular orbital theory and matrix isolation infrared spectroscopy in the study of molecular interactions. *Struct. Chem.* **1992,** 3, (2), 75-93.
7. Wuelfert, S.; Herren, D.; Leutwyler, S., Supersonic jet CARS spectra of small water clusters. *J. Chem. Phys.* **1987,** 86, (6), 3751-3753.
8. Amos, R. D., Structures, harmonic frequencies and infrared intensities of the dimers of H2O and H2S. *Chem. Phys.* **1986,** 104, (1), 145-151.
9. Bentwood, R. M.; Barnes, A. J.; Orville-Thomas, W. J., Studies of intermolecular interactions by matrix isolation vibrational spectroscopy: Self-association of water. *J. Mol. Spectrosc.* **1980,** 84, (2), 391-404.
10. Herzberg, G., *Electronic spectra and electronic structure of polyatomic molecules*. Van Nostrand: New York, 1966.
11. Kwei, G. H.; Curl, R. F., Microwave Spectrum of O18 Formic Acid and Structure of Formic Acid. *J. Chem. Phys.* **1960,** 32, (5), 1592-1594.
12. Miliordos, E.; Xantheas, S. S., On the validity of the basis set superposition error and complete basis set limit extrapolations for the binding energy of the formic acid dimer. *The Journal of Chemical Physics* **2015,** 142, (9), 094311.
13. Derissen, J. L., A reinvestigation of the molecular structure of acetic acid monomer and dimer by gas electron diffraction. *J. Mol. Struct.* **1971,** 7, (1), 67-80.
14. Nagy, P. I., The syn–anti equilibrium for the COOH group reinvestigated. Theoretical conformation analysis for acetic acid in the gas phase and in solution. *Comput. Theor. Chem.* **2013,** 1022, 59-69.
15. Nagy, P. I.; Smith, D. A.; Alagona, G.; Ghio, C., Ab initio studies of free and monohydrated carboxylic acids in the gas phase. *J. Phys. Chem.* **1994,** 98, (2), 486-493.
16. Maçôas, E. M. S.; Lundell, J.; Pettersson, M.; Khriachtchev, L.; Fausto, R.; Räsänen, M., Vibrational spectroscopy of cis- and trans-formic acid in solid argon. *J. Mol. Spectrosc.* **2003,** 219, (1), 70-80.
17. NIST, Computational Chemistry Comparison and Benchmark DataBase Release 22 (May 2022) Standard Reference Database 101 NIST: 2022.
18. Bertie, J. E.; Michaelian, K. H., The Raman spectra of gaseous formic acid -h2 and -d2. *J. Chem. Phys.* **1982,** 76, (2), 886-894.
19. Maréchal, Y., IR spectra of carboxylic acids in the gas phase: A quantitative reinvestigation. *J. Chem. Phys.* **1987,** 87, (11), 6344-6353.
20. Kelemen, A. K.; Luber, S., On the vibrations of formic acid predicted from first principles. *Phys. Chem. Chem. Phys.* **2022,** 24, (46), 28109-28120.
21. Tew, D. P.; Mizukami, W., Ab Initio Vibrational Spectroscopy of cis- and trans-Formic Acid from a Global Potential Energy Surface. *J. Phys. Chem. A* **2016,** 120, (49), 9815-9828.

22. Olbert-Majkut, A.; Ahokas, J.; Lundell, J.; Pettersson, M., Raman spectroscopy of acetic acid monomer and dimers isolated in solid argon. *J. Raman Spectrosc.* **2011,** 42, (8), 1670-1681.
23. Almenningen, A.; Bastiansen, O.; Motzfeldt, T., A reinvestigation of the structure of monomer and dimer formic acid by gas electron diffraction technique. *Acta Chem. Scand.* **1969,** 23, 2848-2864.
24. Kalescky, R.; Kraka, E.; Cremer, D., Accurate determination of the binding energy of the formic acid dimer: The importance of geometry relaxation. *The Journal of Chemical Physics* **2014,** 140, (8), 084315.
25. Olbert-Majkut, A.; Ahokas, J.; Lundell, J.; Pettersson, M., Raman spectroscopy of formic acid and its dimers isolated in low temperature argon matrices. *Chem. Phys. Lett.* **2009,** 468, (4), 176-183.
26. Qu, C.; Bowman, J. M., High-dimensional fitting of sparse datasets of CCSD(T) electronic energies and MP2 dipole moments, illustrated for the formic acid dimer and its complex IR spectrum. *J. Chem. Phys.* **2018,** 148, (24), 241713.
27. Krishnakumar, P.; Maity, D. K., Theoretical studies on dimerization vs. microhydration of carboxylic acids. *Comput. Theor. Chem.* **2017,** 1099, 185-194.
28. Takatani, T.; Hohenstein, E. G.; Malagoli, M.; Marshall, M. S.; Sherrill, C. D., Basis set consistent revision of the S22 test set of noncovalent interaction energies. *J Chem Phys.* **2010,** 132, 144104.
